# Heat shock protein 90 inhibitors overcome the resistance to Fms-like tyrosine kinase 3 inhibitors in acute myeloid leukemia

**DOI:** 10.18632/oncotarget.26045

**Published:** 2018-09-28

**Authors:** Kazuhiro Katayama, Kohji Noguchi, Yoshikazu Sugimoto

**Affiliations:** ^1^ Division of Chemotherapy, Faculty of Pharmacy, Keio University, Tokyo, Japan

**Keywords:** HSP90 inhibitor, FLT3, quizartinib, acute myeloid leukemia, drug resistance

## Abstract

Internal tandem duplication (ITD) in Fms-like tyrosine kinase 3 (FLT3) is frequently observed in acute myeloid leukemia (AML). Quizartinib, gilteritinib, and midostaurin are inhibitors against FLT3-ITD that have good efficacy for FLT3-ITD-positive AML patients. Long-term administration leads to drug resistance through acquired tyrosine kinase domain (TKD) mutations in FLT3-ITD, such as N676K, F691L, D835V, and Y842C. Here, our screen to detect inhibitors capable of overcoming resistance to FLT3 inhibitors identified heat shock protein (HSP) 90 inhibitors as potential candidates. Although Ba/F3 cells expressing FLT3-ITD with TKD mutations (Ba/F3-ITD+N676K, Ba/F3-ITD+F691L, Ba/F3-ITD+D835V, and Ba/F3-ITD+Y842C) showed various resistance patterns to FLT3 inhibitors compared with Ba/F3-ITD cells that express FLT3-ITD lacking TKD mutations, they were more sensitive to HSP90 inhibitors than Ba/F3 cells. Notably, the Ba/F3-ITD+D835V cells were the most sensitive to HSP90 inhibitors. Treatment with HSP90 inhibitors downregulated FLT3 and its downstream signaling and induced G1 arrest followed by apoptosis in Ba/F3-ITD+N676K, Ba/F3-ITD+F691L, Ba/F3-ITD+Y842C, and especially Ba/F3-ITD+D835V cells at lower concentrations compared with Ba/F3-ITD cells. The downregulation of FLT3-ITD+D835V was caused by rapid proteolysis in autophagy. Similar results were also observed in the quizartinib-resistant MV4-11 cells, QR1 and QR2, which were established by culturing cells in the presence of quizartinib and harbored FLT3-ITD+D835H and FLT3-ITD+D835V, respectively, in a single allele. Interestingly, the efficacies of HSP90 inhibitors in QR cells are reversely correlated with that of quizartib, but not to gilteritinib and midostaurin. Collectively, HSP90 inhibitors are good candidates to overcome drug resistance in AML with various FLT3-ITD TKD mutations.

## INTRODUCTION

Acute myeloid leukemia (AML) is a relatively rare cancer, accounting for 1.8% of all cancer deaths in 2017; however, it has a poor prognosis, with only 27.4% survival after 5 years based on 2008–2014 data in the United States [1]. AML is caused by the replacement of normal bone marrow with leukemia cells generated by the growth of abnormal white blood cells. Gradual mutations have been reported to occur during AML cell production from hematopoietic stem cells, in a manner similar to the multistep carcinogenesis model for colon and lung cancers [2, 3]. The first target of the AML multi-mutations is *DNA methyltransferase 3A* (*DNMT3A*), which is followed by mutations in the *Fms-like tyrosine kinase 3* (*FLT3*), *nucleophosmin 1* (*NPM1*), and/or *isocitrate dehydrogenase 1* (*IDH1*) genes. Dovey *et al.* demonstrated that mutations of either *NPM1* or *FLT3* alone or both together caused AML in around 50% or 100%, respectively, of knock-in mice [4]. These mutations highly correlate with the generation of AML. Many chromosomal abnormalities are also observed in AML and produce molecular alterations by chromosome translocations, such as *Runt-related transcription factor* (*RUNX*) *1*–*RUNX1T1*, *Core-binding factor subunit beta* (*CBFB*)–*myosin heavy chain 11* (*MYH11*), *promyelocytic leukemia* (*PML*)–*retinoic acid receptor alpha* (*RARA*), and *mixed lineage leukemia* (*MLL*) fusions with various genes [5]. In addition, DNA methylation and histone modifications significantly contribute to leukemogenesis in AML. Thus, gene mutations, chromosome translocations, and epigenetic modifications are genetically noteworthy for understanding this malignancy.

FLT3 is a receptor tyrosine kinase that regulates several growth signaling pathways, including the signal transducer and activator of transcription 5 (STAT5)–PIM1, phosphoinositide 3-kinase (PI3K)–AKT, and MAPK pathways [6]. In FLT3 wildtype (WT) cells, FLT3 ligand promotes dimerization and activation of FLT3 by self-phosphorylation, resulting in the activation of downstream signaling. In contrast, a 30-base-pair insertion called an internal tandem duplication (ITD) in the *FLT3* gene prompts self-dimerization followed by self-activation, independently of FLT3 ligand [7]. Activating mutations in the tyrosine kinase domain (TKD) of FLT3 are also observed in AML patients. FLT3 mutations, including both ITD and TKD mutations, are detected in 25–30% of AML patients [8]. These FLT3 mutations are driver oncogenes for AML progression, and, thus, they are good molecular targets for treating AML.

Various FLT3 inhibitors are currently under development, and midostaurin (PKC412, Novartis) was approved in 2017 as a first-generation inhibitor for FLT3-ITD- and FLT3-TKD-positive AML in the United States [6, 9–11]. Quizartinib (AC220, Daiichi Sankyo) and gilteritinib (ASP2215, Astellas) are FLT3-specific inhibitors classified as potent second-generation drugs [6, 11–13] and currently under clinical consideration for use in FLT3-ITD-positive AML patients. In the Phase II trial of quizartinib, it improved overall survival in approximately 50% of AML patients [14, 15], but its long-term administration produces AML recurrence, in a part, with quizartinib resistance-conferring mutations of FLT3-ITD. These mutations occur on F691 in the gatekeeper region of FLT3 and on D835, I836, and Y842 in the activation loop region [16–19]. They have been reported to confer the hyper-resistance to quizartinib with lost affinity [20, 21], similar to other tyrosine kinase inhibitors such as imatinib, which induces T315I, M351T, and E355G of BCR-ABL1 [22, 23], and crizotinib, which induces L1196M and C1156Y of ALK [24]. The expression of resistance mutations causes serious problems in clinical settings, so cancer chemotherapies are needed to overcome these drug resistances.

Here, we screened 50 small molecule inhibitors using Ba/F3 cells transfected with FLT3-ITD (Ba/F3-ITD) and those carrying FLT3 inhibitor resistance-conferring mutations (N676K, F691L, D835V, or Y842C) to explore candidates for overcoming the resistance to FLT3 inhibitors, and we identified heat shock protein 90 (HSP90) inhibitors as the best candidates. Parallel results were observed in quizartinib-resistant AML cell lines established from MV4-11 cells that harbored D835H or D835V mutations in FLT3-ITD. Collectively, HSP90 inhibitors show efficacy against FLT3-ITD-positive AML cells, and their efficacies inversely correlates with the efficacy of quizartinib.

## RESULTS

### Establishment of FLT3-ITD transfectants and drug screening

**Figure 1:**
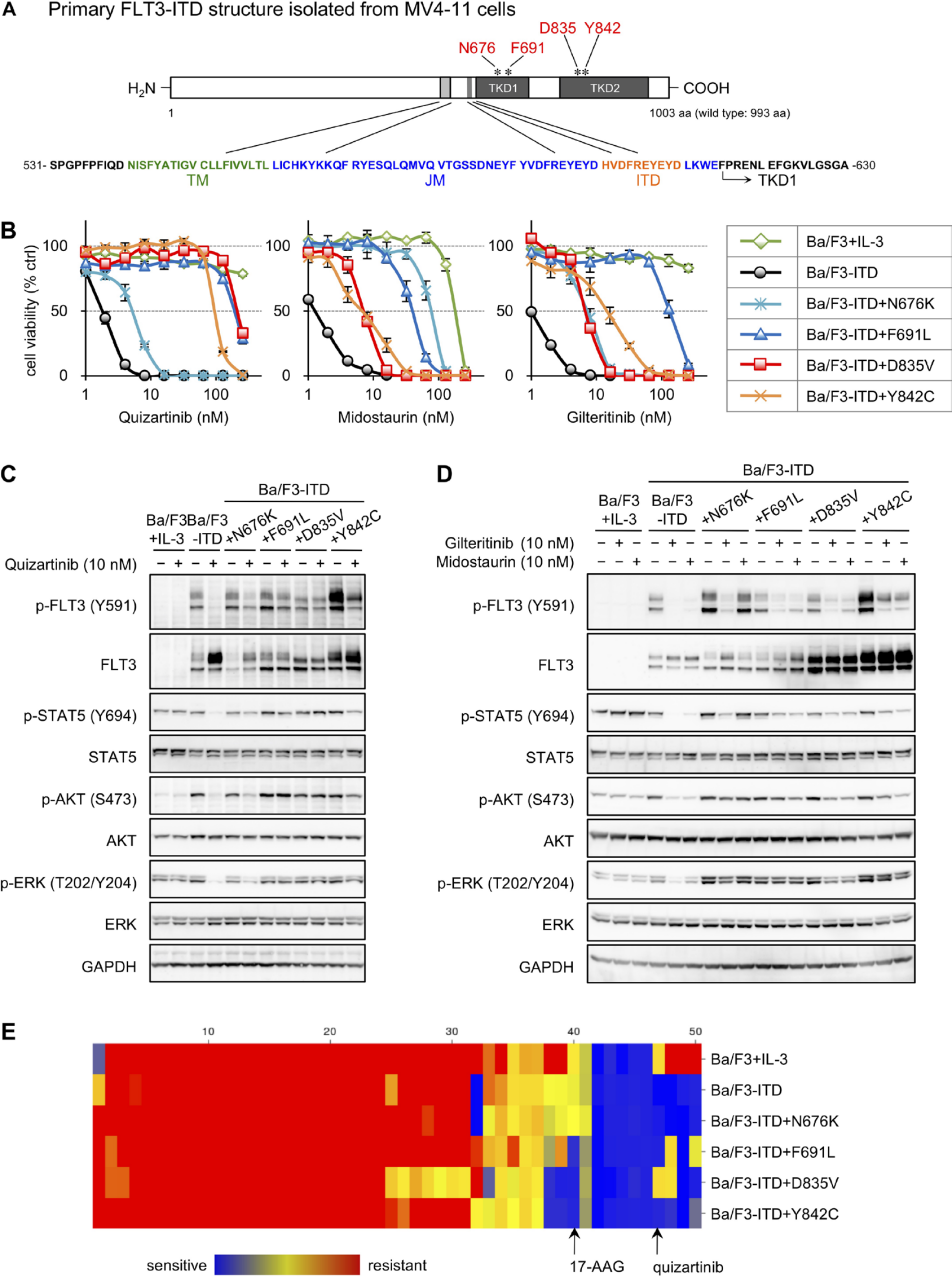
Establishment of FLT3-ITD and TKD mutant transfectants and drug screening (**A**) Schematic primary structure of FLT3-ITD isolated from MV4-11 cells. The positions of N676 and F691 lie on tyrosine kinase domain (TKD) 1, and those of D835 and Y842 are on TKD2. TM, transmembrane domain; JM, juxtamembrane domain; ITD, internal tandem duplication. (**B**) Cells were treated with increasing concentrations of quizartinib, midostaurin, or gilteritinib (1–256 nM) for 4 days, and cell viabilities were determined by WST-8 assay. The viabilities of drug-treated cells relative to those of untreated cells were calculated and are shown here as the mean ± SD from three independent experiments. (**C** and **D**) Cells were treated with or without 10 nM quizartinib (**C**), gilteritinib, or midostaurin (**D**) for 6 h, and immunoblotting using the indicated antibodies was performed. (**E**) Cells were treated with 10–1000 nM concentrations of various inhibitors, listed in Supplementary Table 1, for 4 days, and cell viabilities were determined by WST-8 assay. The clustered image map was created at the CIMminor site using the average IC_50_ values from triplicated experiments, as described in the Methods section.

*FLT3*-ITD cDNA was isolated from a MV4-11 cDNA library, and TKD mutant cDNAs were generated by mutagenesis based on FLT3-ITD. The N676K and F691L mutations lie within TKD1 of FLT3-ITD, and the D835V and Y842C mutations are within TKD2 (Figure 1A). Ba/F3 cells were stably transfected with either FLT3-ITD or one of each TKD mutant, and the resulting cells were named Ba/F3-ITD, Ba/F3-ITD+N676K, Ba/F3-ITD+F691L, Ba/F3-ITD+D835V, and Ba/F3-ITD+Y842C. As Ba/F3 cells require mouse interleukin-3 (mIL-3) for their proliferation, all experiments using parental Ba/F3 cells were performed in the presence of mIL-3. In contrast, the growth of the transfectants depends on only self-activated FLT3 signaling, so the transfectants were cultured without any cytokine supplementation.

We first examined sensitivity to FLT3 inhibitors by cell growth inhibition assays (Figure 1B and Table 1) and immunoblotting (Figure 1C and 1D). In growth inhibition assays, Ba/F3-ITD+D835V and Ba/F3-ITD+Y842C cells showed quizartinib resistance with IC_50_ values of 217 nM and 101 nM, respectively, compared with Ba/F3-ITD cells (IC_50_ value: 1.85 nM) (Figure 1B, left, and Table 1). In contrast, Ba/F3-ITD+N676K cells, which are reportedly resistant to midostaurin (Figure 1B, middle, and Table 1) but not to quizartinib [25, 26], showed minimal quizartinib resistance (Figure 1B, left, and Table 1). Ba/F3-ITD+N676K, Ba/F3-ITD+D835V, and Ba/F3-ITD+Y842C cells showed approximately 10-fold resistance to gilteritinib compared with Ba/F3-ITD cells (Figure 1B, right and Table 1). Furthermore, Ba/F3-ITD+F691L cells showed over 100-fold resistance to both quizartinib and gilteritinib and over 30-fold resistance to midostaurin compared with Ba/F3-ITD cells (Figure 1B and Table 1). Immunoblotting (Figure 1C) revealed that quizartinib inhibited FLT3 phosphorylation and its downstream signaling, monitored by phosphorylated STAT5, AKT, and extracellular signal-regulated kinase (ERK), in Ba/F3-ITD and Ba/F3-ITD+N676K cells but not in Ba/F3-ITD+F691L and Ba/F3-ITD+D835V cells. Phosphorylation of these kinases was partially downregulated in Ba/F3-ITD+Y842C cells, in agreement with previous reports [20, 21]. As shown in Figure 1D, gilteritinib downregulated phosphorylated FLT3 and STAT5 in all transfectants, but its abilities to reduce phosphorylated AKT and ERK levels were limited in TKD mutants. Similar results were obtained in cells treated with midostaurin except for Ba/F3-ITD+N676K cells, which are resistant cells against this inhibitor. These results indicate that our cell lines were successfully established and useful for further experiments and that TKD mutations in FLT3-ITD confer resistances to FLT3 inhibitors.

**Table 1:** Cell growth-inhibition profile for FLT3 inhibitors in Ba/F3 transfectants

	Quizartinib		Gilteritinib		Midostaurin	
	IC_50_ (nM)	RR (vs ITH)	IC_50_ (nM)	RR (vs ITH)	IC_50_ (nM)	RR (vs ITD)
Ba/F3+IL-3	>256	–	>256	–	184	–
Ba/F3-ITD	1.85	1.0	0.973	1.0	1.34	1.0
Ba/F3-ITD+N676K	5.39	2.9	7.34	7.5	80.2	60
Ba/F3-ITD+F691L	198	110	132	140	41.6	31
Ba/F3-ITD+D835V	217	120	6.99	7.2	7.25	5.4
Ba/F3-ITD+Y842C	101	54	14.8	15	6.94	5.2

The corresponding data are presented in Figure 1B. The IC_50_ values were determined from the growth inhibition curves. The relative resistance (RR) was calculated by dividing the IC_50_ values of each cell line by that of Ba/F3-ITD cells.

Next, drug screening using 50 inhibitors (listed in Supplementary Table 1) was performed via cell growth inhibition assays to find effective inhibitors against the proliferation of Ba/F3-ITD+F691L, Ba/F3-ITD+D835V, and Ba/F3-ITD+Y842C cells (Figure 1E). A clustered image map using the IC_50_ values from three independent experiments shows that several inhibitors specifically suppressed proliferation of these cell lines. Among them, we focused on 17-allylamino-17-demethoxygeldanamycin (17-AAG) for further experiments because it inhibited the growth of Ba/F3-ITD+F691L, Ba/F3-ITD+D835V, and Ba/F3-ITD+Y842C cells in lower concentrations compared with Ba/F3-ITD cells.

### Ba/F3-ITD+D835V cells are the TKD mutants most sensitive to HSP90 inhibitors

**Figure 2:**
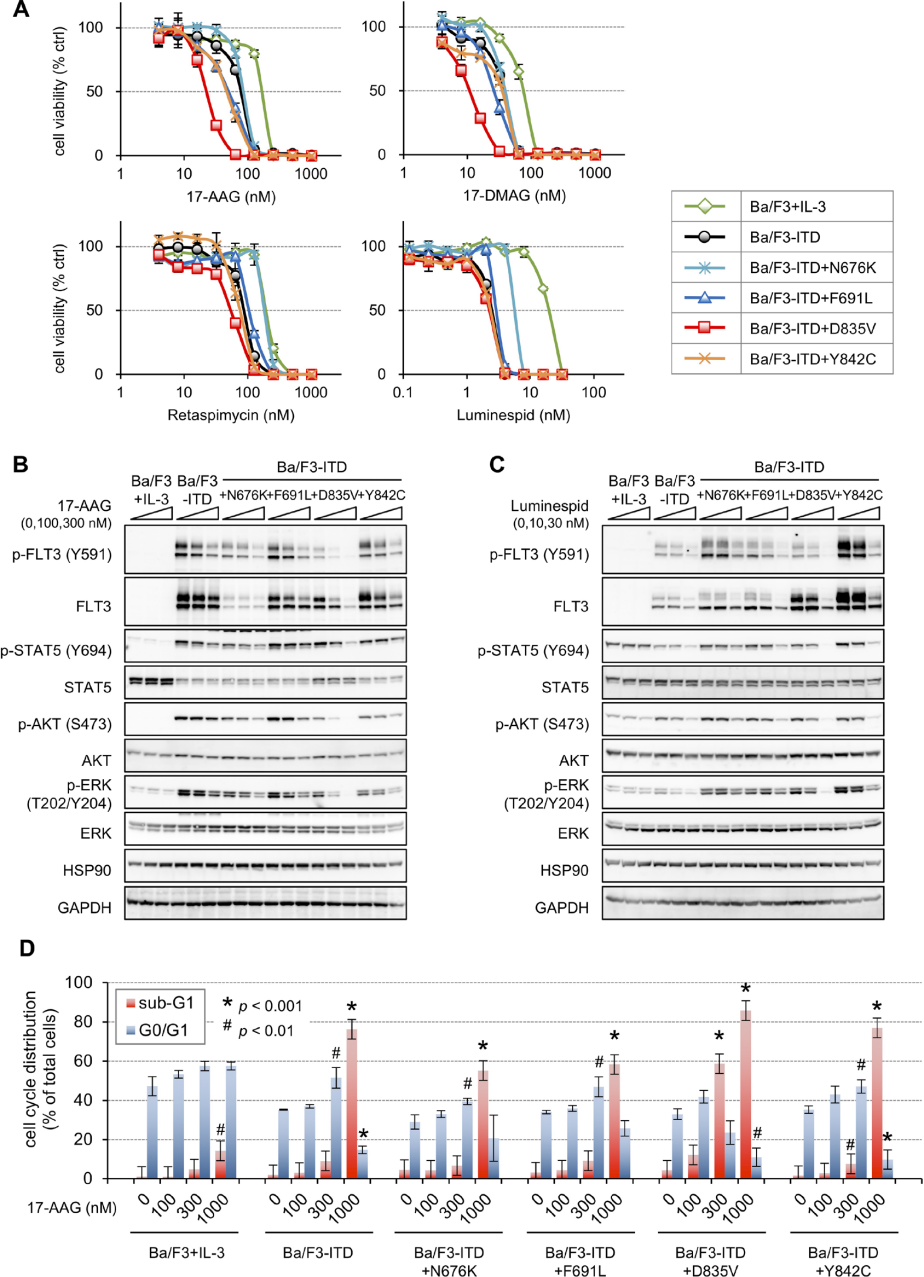
Sensitivities to HSP90 inhibitors in the FLT3-ITD and TKD mutant transfectants (**A**) Cells were treated with increasing concentrations of 17-AAG (upper left; 4–1024 nM), 17-DMAG (upper right; 4–1024 nM), retaspimycin (lower left; 4–1024 nM), or luminespid (lower right; 0.125–32 nM) for 4 days, and the resulting cell viabilities were determined by WST-8 assay. The viabilities of 17-AAG- or 17-DMAG-treated cells relative to those of untreated cells were calculated and are presented here as the mean ± SD from three independent experiments. (**B** and **C**) Cells were treated with or without 100 or 300 nM 17-AAG (**B**) or 10 or 30 nM luminespid (**C**) for 6 h, and immunoblotting using the indicated antibodies was performed. (**D**) Cells were treated with 100–1000 nM 17-AAG for 24 h, and, after staining the cells with PI, the cell cycle ploidy patterns were analyzed by FACS. Each population was calculated using CellQuest software. The ratios of sub-G1 (red) and G0/G1 (blue) phases are presented in the bar graph as the mean ± SD from three independent experiments; statistical analyses were performed with two-tailed Student's *t*-tests. Typical corresponding raw data are shown in Supplementary Figure 3.

Additional cell growth inhibition assays were performed to investigate the details of the 17-AAG effects (Figure 2A, upper left). Lower 17-AAG concentrations were needed to suppress growth of the transfectants compared with the Ba/F3 cells. The relative 17-AAG resistances, which were calculated by dividing the IC_50_ values for each transfectant by that for Ba/F3 cells, ranged from 0.14–0.52 (Table 2). Ba/F3-ITD+F691L and Ba/F3-ITD+Y842C cells showed 17-AAG sensitivities approximately 1.5 times higher compared with Ba/F3-ITD and Ba/F3-ITD+N676K cells, and Ba/F3-ITD+D835V cells were three times more sensitive to 17-AAG than Ba/F3-ITD cells. Like 17-AAG, other HSP90 inhibitors, 17-dimethylaminoethylamino-17-demethoxygeldanamycin (17-DMAG), retaspimycin (hydroquinone derivative of 17-AAG), and luminespid (resorcinylic isoxazole amide), also suppressed growth of the transfectants at lower concentrations compared with Ba/F3 cells (Figure 2A and Table 2). Among these HSP90 inhibitors, luminespid suppressed proliferation of the transfectants most specifically compared with Ba/F3 cells.

**Table 2:** Cell growth-inhibition profile for HSP90 inhibitors in Ba/F3 transfectants

	17-AAG		17-DMAG		Retaspimycin		Luminespid	
	IC_50_ (nM)	RR (vs Ba/F3)	IC_50_ (nM)	RR (vs Ba/F3)	IC_50_ (nM)	RR (vs Ba/F3)	IC_50_ (nM)	RR (vs Ba/F3)
Ba/F3+IL-3	176	1.0	78.2	1.0	204	1.0	20.1	1.0
Ba/F3-ITD	81.6	0.46	38.7	0.49	91.7	0.45	2.59	0.13
Ba/F3-ITD+N676K	90.6	0.52	41.2	0.53	193	0.95	5.98	0.30
Ba/F3-ITD+F691L	52.0	0.30	27.2	0.35	109	0.54	3.06	0.15
Ba/F3-ITD+D835V	23.8	0.14	11.4	0.15	55.7	0.27	2.32	0.12
Ba/F3-ITD+Y842C	48.8	0.28	35.8	0.46	79.3	0.39	2.44	0.12

The corresponding data are presented in Figure 2A. The IC_50_ values were determined from the growth inhibition curves. The relative resistance (RR) was calculated by dividing the IC_50_ values of each cell line by that of Ba/F3 cells.

To confirm that Ba/F3-ITD+D835V cells are particularly sensitive to 17-AAG, we independently reestablished Ba/F3-ITD+D835V cells (named Ba/F3-ITD+D835V-2 cells) and performed growth inhibition assays (Supplementary Figure 1). Comparable results were obtained in two independent Ba/F3-ITD+D835V cell lines, which both had 17-AAG sensitivities approximately three times higher compared with Ba/F3-ITD cells. Immunoblotting was performed to examine FLT3 signaling in cells treated with 100 and 300 nM 17-AAG for 6 h (Figure 2B). These conditions were determined based on the downregulation of phosphorylated FLT3, STAT5, AKT, and ERK that was observed at 100 nM 17-AAG in Ba/F3-ITD+D835V cells and at 300 nM 17-AAG in Ba/F3-ITD and Ba/F3-ITD+N676K cells as well as the complete suppression observed in all cell lines by 1000 nM 17-AAG (Supplementary Figure 2). In parallel with the growth inhibition assay results, downregulation of FLT3 and its downstream signaling in Ba/F3-ITD+D835V cells occurred at lower 17-AAG concentrations compared with other transfectants. The signaling in Ba/F3-ITD+D835V cells was completely downregulated at a 17-AAG concentration of 300 nM, whereas the signaling in other transfectants was only partially suppressed in the same 17-AAG concentrations. 17-AAG did not affect the signaling in Ba/F3 cells at the tested concentrations, although it downregulated the phosphorylation levels of STAT5, AKT, and ERK in Ba/F3 cells treated with 1000 nM for 6 h or with ≥ 300 nM for 24 h (data not shown). An experiment similar to the one shown in Figure 2B was performed in cells treated with 10 or 30 nM luminespid for 6 h (Figure 2C). Again, downregulation of FLT3 and its downstream signaling, including phosphorylated STAT5, AKT, and ERK, was the most obvious in Ba/F3-ITD+D835V cells compared with the other tested cell lines. We then examined cell cycle progression in cells treated with 100–1000 nM 17-AAG for 24 h (Figure 2D and Supplementary Figure 3). 17-AAG concentrations of ≥300 nM induced cell cycle arrest at G0/G1 phase in Ba/F3 cells, but the appearance of a sub-G1 population was limited at all tested concentrations. G1 arrest was induced at 100 nM 17-AAG in Ba/F3-ITD+D835V and Ba/F3-ITD+Y842C cells and at 300 nM in the other transfectants. A significant increase in the sub-G1 population was observed in Ba/F3-ITD+D835V cells at 17-AAG concentrations of ≥300 nM and in the other transfectants at 1000 nM.

**Figure 3:**
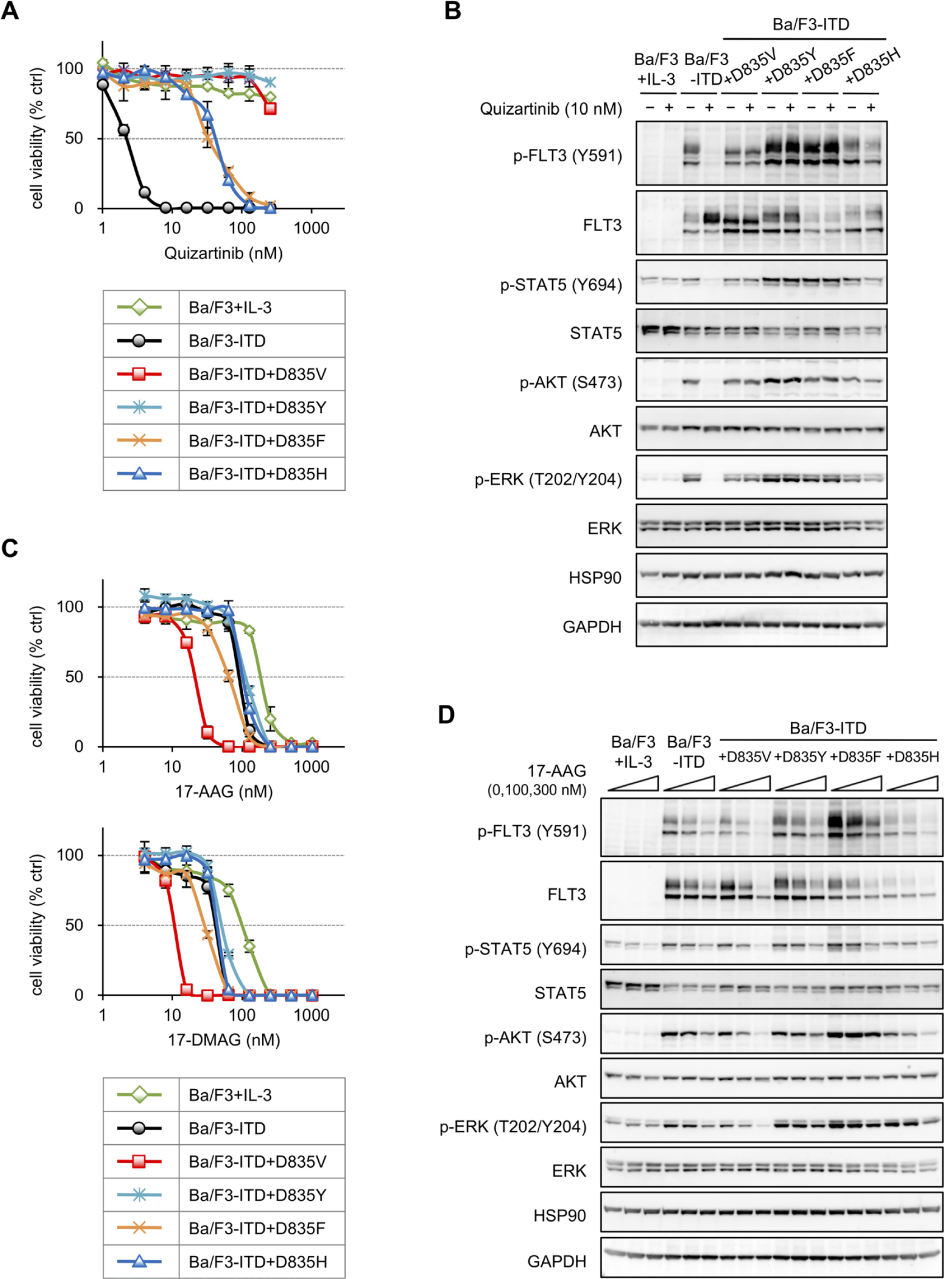
Sensitivities to HSP90 inhibitors in Ba/F3 cells expressing various amino acid substitutions of D835 on FLT3-ITD (**A**) Cells were treated with the increasing concentrations of quizartinib (1–256 nM) for 4 days, and the resulting cell viabilities were determined by WST-8 assay. The data are presented here as the mean ± SD from three independent experiments. (**B**) Cells were treated with or without 10 nM quizartinib for 6 h, and lysates of these cells were subjected to immunoblotting using the indicated antibodies. (**C**) Cells were treated with increasing concentrations of 17-AAG (upper; 4–1024 nM) or 17-DMAG (lower; 4–1024 nM) for 4 days, after which cell growth inhibition assays were performed as described in (**A**). (**D**) Cells were treated with or without 100 or 300 nM 17-AAG for 6 h, and lysates of these cells were subjected to immunoblotting using the indicated antibodies.

Several substitutions of D835 in FLT3 have been reported previously [16–21]. As our above results showed that HSP90 inhibitors strongly suppressed Ba/F3-ITD+D835V cell proliferation, we examined the effect of 17-AAG on cells harboring other D835 substitutions (i.e., Ba/F3-ITD+D835Y, Ba/F3-ITD+D835F, and Ba/F3-ITD+D835H cells). Before this experiment, we confirmed the quizartinib resistance of these cell lines. All D835 mutants showed higher quizartinib resistance than Ba/F3-ITD cells as observed via growth inhibition assays (Figure 3A and Table 3) and immunoblotting (Figure 3B). Growth inhibition assays using 17-AAG (Figure 3C, top, and Table 4) revealed that all these mutants had higher 17-AAG sensitivities compared with Ba/F3 cells, but the sensitivities of Ba/F3-ITD+D835Y and Ba/F3-ITD+D835H cells were similar to that of Ba/F3-ITD cells. Although 17-AAG was more efficacious against Ba/F3-ITD+D835F cells than Ba/F3-ITD cells, the efficacy was three times weaker than that against Ba/F3-ITD+D835V cells. Similar results were obtained in the growth inhibition assay using 17-DMAG (Figure 3C, bottom, and Table 4). Immunoblotting (Figure 3D) revealed that phosphorylated FLT3, AKT, and ERK levels were high in Ba/F3-ITD+D835F cells, and 17-AAG treatment downregulated the levels of phosphorylated FLT3 and STAT5 but not of phosphorylated AKT and ERK. 17-AAG-mediated downregulation of phosphorylated FLT3, STAT5, and AKT was also observed in Ba/F3-ITD+D835Y and Ba/F3-ITD+D835H cells; however, the responses were weaker than that in Ba/F3-ITD+D835V cells. These results suggest that HSP90 inhibitors specifically suppress FLT3-ITD+D835V rather than other D835 substitutions.

**Table 3:** Cell growth-inhibition profile for FLT3 inhibitors in FLT3-ITD-D835 mutant cells

	Quizartinib	
	IC_50_ (nM)	RR (vs ITD)
Ba/F3+IL-3	>256	–
Ba/F3-ITD	2.27	1.0
Ba/F3-ITD+D835V	>256	>110
Ba/F3-ITD+D835Y	>256	>110
Ba/F3-ITD+D835F	33.2	15
Ba/F3-ITD+D835H	43.6	19

The corresponding data are presented in Figure 3A. The IC_50_ values were determined from the growth inhibition curves. The relative resistance (RR) was calculated by dividing the IC_50_ values of each cell line by that of Ba/F3-ITD cells.

**Table 4:** Cell growth-inhibition profile for HSP90 inhibitors in FLT3-ITD-D835 mutant cells

	17-AAG		17-DMAG	
	IC_50_ (nM)	RR (vs Ba/F3)	IC_50_ (nM)	RR (vs Ba/F3)
Ba/F3+IL-3	195	1.0	104	1.0
Ba/F3-ITD	96.2	0.49	43.4	0.42
Ba/F3-ITD+D835V	22.1	0.11	11.3	0.11
Ba/F3-ITD+D835Y	116	0.59	53.6	0.51
Ba/F3-ITD+D835F	65.1	0.33	29.6	0.28
Ba/F3-ITD+D835H	107	0.55	46.5	0.45

The corresponding data are presented in Figure 3C. The IC_50_ values were determined from the growth inhibition curves. The relative resistance (RR) was calculated by dividing the IC_50_ values of each cell line by that of Ba/F3 cells.

### 17-AAG induces lysosomal protein degradation of FLT3-ITD+D835V

**Figure 4:**
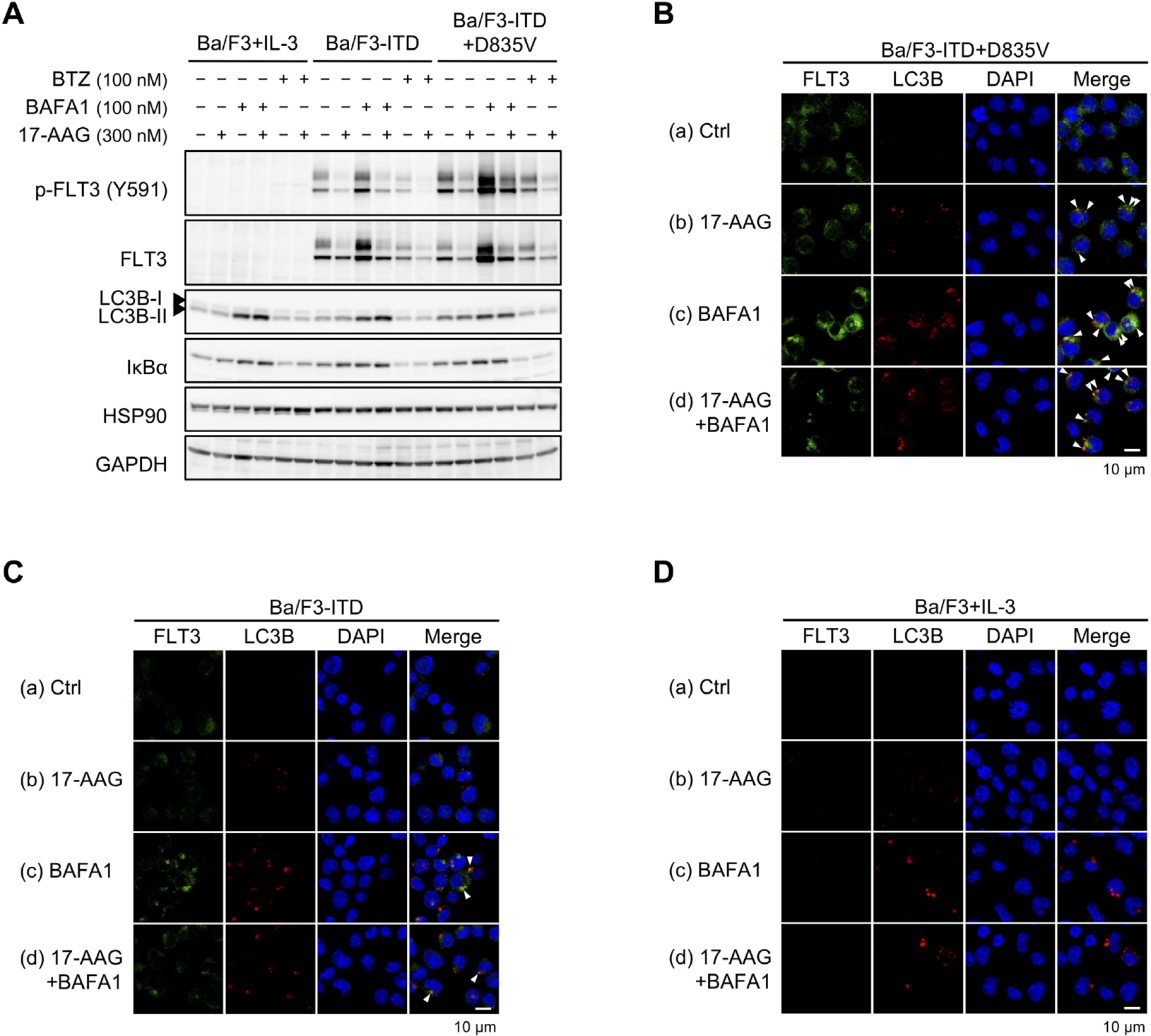
Lysosomal degradation of FLT3-ITD+D835V by 17-AAG Cells were treated with or without 300 nM 17-AAG combined with 100 nM bafilomycin A1 (BAFA1) or bortezomib (BTZ) for 6 h. (**A**) Immunoblotting using the indicated antibodies was performed. (**B**–**D**) Cells were stained with anti-FLT3 (green signals) and anti-LC3B (red signals) antibodies. Nuclei were counterstained with DAPI. Arrowheads indicate FLT3 merged with LC3B (yellow signals).

To examine whether the rapid FLT3 downregulation observed following 17-AAG treatment is caused by protein degradation, cells were treated with 17-AAG combined with bafilomycin A1 (BAFA1), an inhibitor of lysosomal protein degradation, or bortezomib (BTZ), a proteasome inhibitor, for 6 h (Figure 4A). The 17-AAG-mediated downregulation of FLT3 expression and phosphorylation was partially suppressed by BAFA1 but not by BTZ. Although the effect of BAFA1 was observed clearly in Ba/F3-ITD+D835V cells, it was limited in Ba/F3-ITD cells. We then performed immunofluorescence staining to examine the localization of FLT3 and LC3B in cells treated with or without 17-AAG and/or BAFA1 for 6 h (Figure 4B–4D). BAFA1-mediated upregulation of FLT3 was observed in Ba/F3-ITD+D835V cells compared with untreated cells (Figure 4Ba vs 4Bc), and many LC3B foci were co-localized with FLT3 in BAFA1-treated cells (Figure 4Bc and 4Bd). Treatment with 17-AAG also induced some LC3B foci, and they were merged with FLT3 in Ba/F3-ITD+D835V cells (Figure 4Bb). In contrast, co-localization of FLT3 with LC3B foci was marginally observed in BAFA1-treated Ba/F3-ITD cells (Figure 4Cc and 4Cd) but not in Ba/F3 cells (Figure 4D). These results suggest that 17-AAG induces rapid lysosomal degradation of FLT3 in Ba/F3-ITD+D835V cells.

### 17-AAG does not affect HSP90 binding to FLT3-ITD

**Figure 5:**
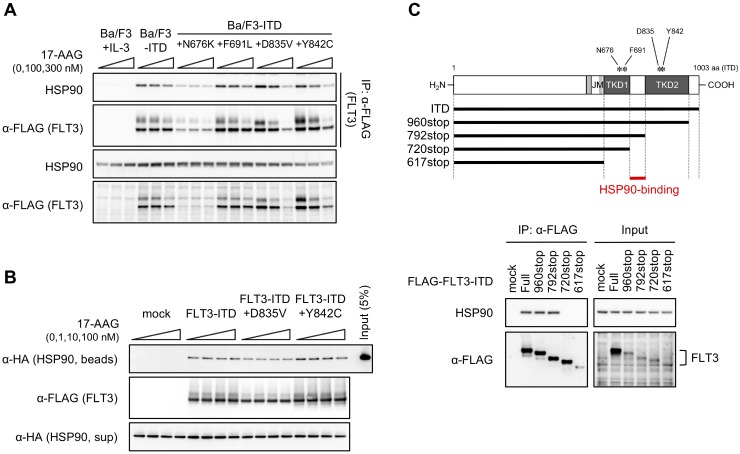
Effect of 17-AAG on FLT3-ITD–HSP90 binding (**A**) Cells were treated with or without 100 or 300 nM 17-AAG for 6 h. FLAG-tagged FLT3-ITD proteins were immunoprecipitated with an anti-FLAG antibody, and the immunoprecipitants were subjected to immunoblotting using anti-HSP90 or anti-FLAG antibodies. (**B**) HA-tagged HSP90 proteins were immunopurified from HEK293 transfectants, and FLAG-tagged FLT3-ITD proteins bound to affinity gels were prepared from Ba/F3 transfectants. Both proteins were mixed and incubated in immunoprecipitation buffer with or without 1–100 nM 17-AAG by rocking overnight at 4° C. The immunoprecipitants were eluted with FLAG peptides and subjected to immunoblotting using anti-HA or anti-FLAG antibodies. (**C**) Schematic of the primary structures of FLT3-ITD deletion mutants (top). JM, juxtamembrane domain; TKD, tyrosine kinase domain. HEK293 cells were transfected with FLAG-tagged FLT3-ITD or the deletion mutants for 24 h. The proteins were immunoprecipitated with an anti-FLAG antibody, and the immunoprecipitants were subjected to immunoblotting using anti-HSP90 or anti-FLAG antibodies (bottom).

As the previous results (Figures 2–4) suggest the possibility that a D835 mutation lessens the binding affinity of FLT3-ITD to HSP90, we examined the protein binding in 17-AAG-treated cells (Figure 5A). Immunoprecipitation–immunoblotting revealed that FLT3-co-precipitated HSP90 levels decreased in all 17-AAG-treated transfectants in parallel with FLT3-ITD expression. As such, among all tested transfectants, the relative co-precipitated HSP90 levels were the lowest in 17-AAG-treated Ba/F3-ITD+D835V cells. Due to different FLT3-ITD expressions among the cells, the effect of 17-AAG on HSP90 binding cannot be determined directly, so *in vitro* pulldown assays were performed in the presence or absence of 17-AAG (Figure 5B). Again, HSP90 co-precipitated with recombinant FLT3-ITD, FLT3-ITD+D835V, and FLT3-ITD+Y842C, and the binding did not change in the presence of 17-AAG. These results suggest that 17-AAG does not affect the FLT3-ITD–HSP90 binding despite destabilizing the quizartinib-resistant FLT3-ITD mutants.

The HSP90-binding site was determined by using various FLT3-ITD deletion mutants (Figure 5C). The region between TKD1 and TKD2 in FLT3-ITD was identified as the binding region based on the finding that HSP90 did not co-precipitate with 617stop and 720stop FLT3-ITD, although co-precipitants were detected in 792stop, 960stop, and full-length FLT3-ITD.

### Quizartinib-resistant MV4-11 cells are more sensitive to HSP90 inhibitors than their parental cells

**Figure 6:**
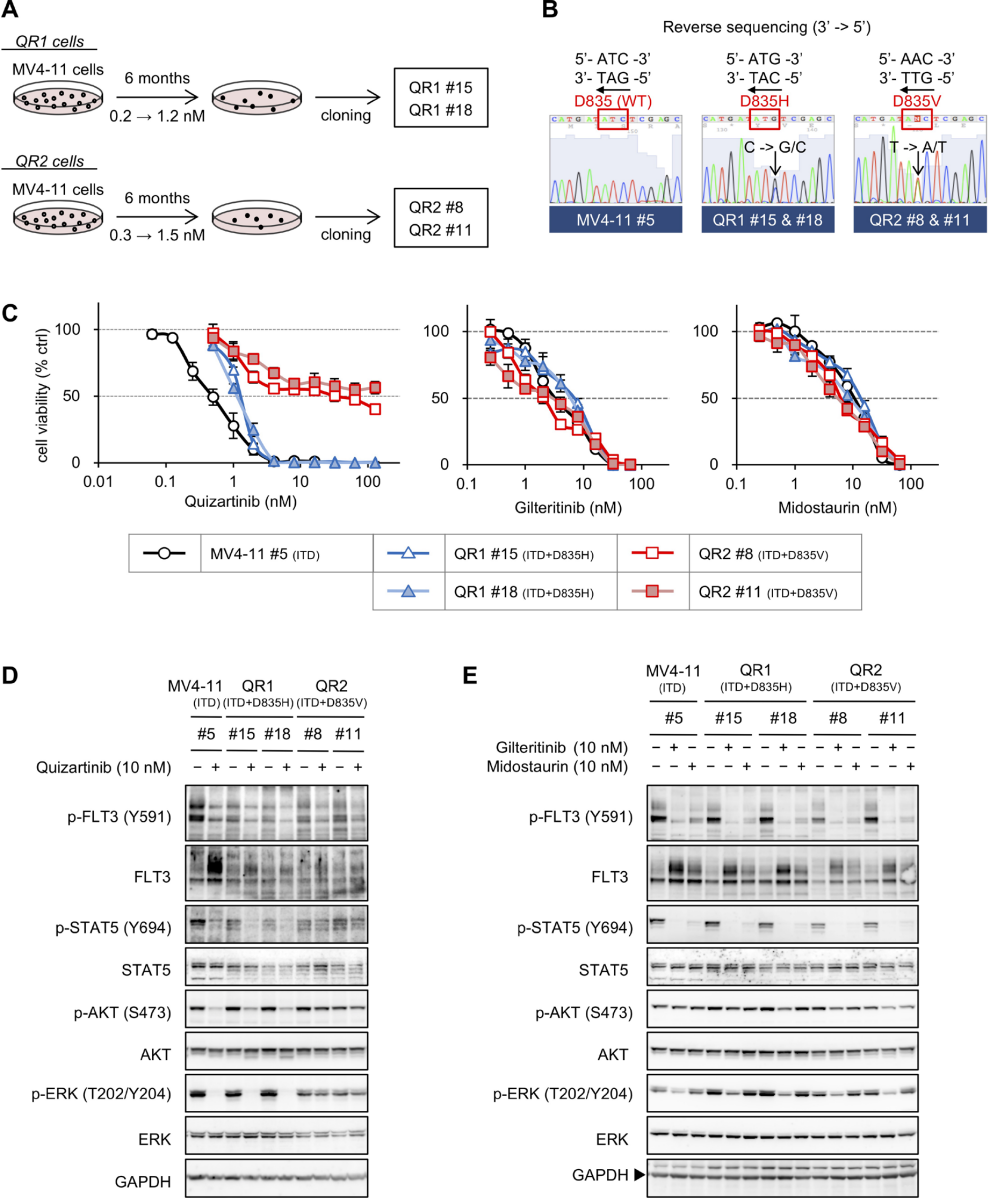
Establishment of quizartinib-resistant MV4-11 cells (**A**) Schematic of the protocol for establishing quizartinib-resistant MV4-11 cells, QR1 and QR2. The QR1 and QR2 cells were independently established from MV4-11 cells cultured under increasing concentrations of quizartinib for 6 months. QR1 #15 and #18 cells were cloned by the limiting dilution method in the presence of 1.2 nM quizartinib, or QR2 #8 and #11 cells were similarly cloned in the presence of 1.5 nM quizartinib. (**B**) Total RNA was isolated from each cell line, and cDNA was synthesized by reverse transcription. Sequencing of the full-length *FLT3* gene was performed after amplification by standard PCR using the cDNA. The raw sequencing data around D835 are shown. (**C**) Cells were treated with increasing concentrations of quizartinib (0.063–16 nM in MV4-11 #5 and 0.5–128 nM in QR1 and QR2 cells), gilteritinib (0.25–64 nM), or midostaurin (0.25–64 nM) for 4 days, and the resulting cell viabilities were determined by WST-8 assay. The data are presented here as the mean ± SD from three independent experiments. (**D** and **E**) Cells were treated with or without 10 nM quizartinib (**D**), gilteritinib, or midostaurin (**E**) for 6 h, and immunoblotting was performed using the indicated antibodies.

QR1 and QR2 cells were independently established from MV4-11 cells, which are FLT3-ITD-positive AML cells, by culturing under increasing concentrations of quizartinib for 6 months, followed by cloning via the limiting dilution method (Figure 6A). Direct sequencing revealed that QR1 #15 and QR1 #18 cells each harbor FLT3-ITD+835H, and QR2 #8 and QR2 #11 cells have FLT3-ITD+835V as one allele and FLT3-ITD+835D (WT) as the other, but no other mutations were observed in the *FLT3* gene (Figure 6B and data not shown). QR1 cells showed quizartinib resistance approximately 2.5 times as high as that of MV4-11 clone #5, and QR2 #8 and QR2 #11 were 73 and >260 times more resistant to quizartinib, respectively, compared with MV4-11 #5 cells (Figure 6C, left and Table 5). In contrast, these quizartinib-resistant cells did not show any resistances to gilteritinib and midostaurin (Figure 6C, middle and right, and Table 5). Quizartinib treatment lowered the FLT3 signaling in both MV4-11 and QR1 cells, but the downregulation of phosphorylated FLT3 and upregulation of FLT3 expression were limited in QR1 cells. In contrast, quizartinib did not change FLT3 signaling in QR2 cells (Figure 6D). A similar experiment using gilteritinib and midostaurin in QR1 and QR2 cells revealed that both inhibitors downregulated the phosphorylation levels of FLT3 and STAT5 but did not change the AKT phosphorylation levels, except in QR2 #11 cells (Figure 6E). At inhibitor concentrations of 10 nM, the downregulation of phosphorylated ERK was observed in all gilteritinib-treated cells but not in any midostaurin-treated cells. These results suggest that QR1 clones are moderately quizartinib-resistant and QR2 clones are hyper-quizartinib-resistant, but they still possess sensitivity to other FLT3 inhibitors.

**Table 5:** Cell growth-inhibition profile for FLT3 inhibitors in quizartinib-resistant MV4-11 cells

	Quizartinib		Gilteritinib		Midostaurin	
	IC_50_ (nM)	RR (vs MV4-11 #5)	IC_50_ (nM)	RR (vs MV4-11 #5)	IC_50_ (nM)	RR (vs MV4-11 #5)
MV4-11 #5	0.492	1.0	3.47	1.0	11.1	1.0
QR1 #15	1.36	2.8	6.15	1.8	14.0	1.3
QR1 #18	1.20	2.4	6.19	1.8	9.09	0.82
QR2 #8	36.1	73	2.11	0.61	6.60	0.59
QR2 #11	>128	>260	3.04	0.88	6.43	0.58

The corresponding data are presented in Figure 6C. The IC_50_ values were determined from the growth inhibition curves. The relative resistance (RR) was calculated by dividing the IC_50_ values of each cell line by that of MV4-11 #5 cells.

**Figure 7:**
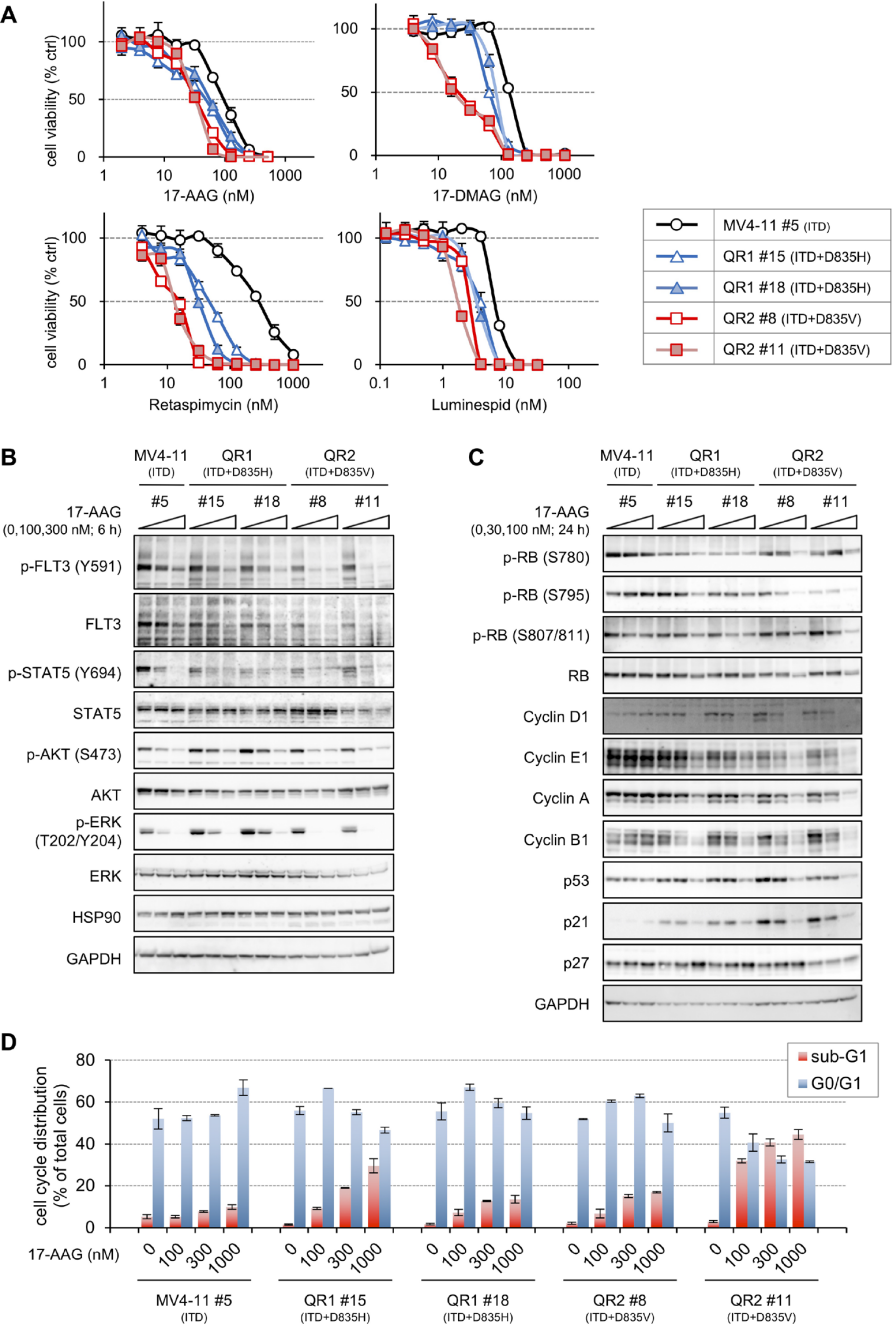
Sensitivities to 17-AAG in QR1 and QR2 cells (**A**) Cells were treated with increasing concentrations of 17-AAG (2–512 nM), 17-DMAG (4–1024 nM), retaspimycin (4–1024 nM), or luminespid (0.125–32 nM) for 4 days, and the resulting cell viabilities were determined by WST-8 assay. The data are presented here as the mean ± SD from three independent experiments. (**B**) Cells were treated with or without 100 or 300 nM 17-AAG for 6 h, and immunoblotting using the indicated antibodies was performed. (**C**) Cells were treated with or without 30 or 100 nM 17-AAG for 24 h, and immunoblotting using the indicated antibodies was performed. (**D**) Cells were treated with 100–1000 nM 17-AAG for 24 h, and, after staining the cells with PI, the cell cycle ploidy patterns were analyzed by FACS. Each population was measured using CellQuest software, and the data are presented in the bar graph as the mean ± SE from two independent experiments. Typical corresponding raw data are shown in Supplementary Figure 5.

We examined sensitivity to HSP90 inhibitors in QR cells via growth inhibition assays (Figure 7A and Table 6). QR1 (#15 and #18) and QR2 (#8 and #11) cells were approximately 2–7 times more sensitive to 17-AAG, 17-DMAG, and luminespid compared with MV4-11 #5 cells. Among the tested HSP90 inhibitors, retaspimycin is the most effective inhibitor of cell proliferation in QR1 and QR2 cells (Figure 7A, lower left). The relative resistance indices of QR1 and QR2 cells were 0.05-0.17 compared with MV4-11 #5 cells (Table 6). Immunoblotting (Figure 7B) revealed that FLT3 signaling in MV4-11 and QR1 cells was downregulated by 17-AAG treatment for 6 h, starting at 100 nM and becoming completely undetectable at 300 nM. However, the degree of the downregulation in QR1 cells was higher than that in MV4-11 cells because the baseline phosphorylation levels of AKT and ERK in QR1 cells were higher than those in MV4-11 cells, and 17-AAG lowered them to the same level in both cell types. In contrast, FLT3 signaling in QR2 cells was completely downregulated by 100 nM 17-AAG. Downregulation of FLT3 and its downstream signals was also observed in QR2 (#8 and #11) cells treated with 30 nM luminespid for 6 h (Supplementary Figure 4).

**Table 6:** Cell growth-inhibition profile for HSP90 inhibitors in quizartinib-resistant MV4-11 cells

	17-AAG		17-DMAG		Retaspimycin		Luminespid	
	IC_50_ (nM)	RR (vs MV4-11 #5)	IC_50_ (nM)	RR (vs MV4-11 #5)	IC_50_ (nM)	RR (vs MV4-11 #5)	IC_50_ (nM)	RR (vs MV4-11 #5)
MV4-11 #5	101	1.0	136	1.0	292	1.0	6.92	1.0
QR1 #15	49.6	0.49	64.0	0.47	48.6	0.17	3.95	0.57
QR1 #18	58.0	0.57	86.3	0.63	31.7	0.11	3.54	0.51
QR2 #8	33.9	0.34	22.1	0.16	15.1	0.052	2.78	0.40
QR2 #11	33.2	0.33	18.7	0.14	13.7	0.047	1.79	0.26

The corresponding data are presented in Figure 7A. The IC_50_ values were determined from the growth inhibition curves. The relative resistance (RR) was calculated by dividing the IC_50_ values of each cell line by that of MV4-11 #5 cells.

The expressions of cell cycle-related proteins were investigated in QR cells treated with 30 or 100 nM 17-AAG for 24 h (Figure 7C). 17-AAG dephosphorylated retinoblastoma protein (RB) and lowered cyclins D1, E1, A, and B1 in only QR1 and QR2 cells. Additionally, downregulations of tumor suppressor p53 and p21 were observed in both QR1 and QR2 cells, and upregulation of p27 was observed in QR1 #15, QR1 #18, and QR2 #8. Ploidy patterns of cells treated with 100–1000 nM 17-AAG for 24 h were also examined by FACS after staining with PI (Figure 7D and Supplementary Figure 5). The G0/G1 phase populations in MV4-11 #5 cells were slightly increased at 1000 nM 17-AAG. The drug increased the G0/G1 phase population followed by the sub-G1 population in a dose-dependent manner in QR1 cells. In QR2 #8 cells, 100 and 300 nM 17-AAG arrested the cell cycle at G1 phase, and the sub-G1 population was increased by >300 nM 17-AAG, whereas the sub-G1 population was remarkably increased by >100 nM 17-AAG in QR2 #11 cells. These results indicate that 17-AAG induces both cell cycle arrest at G1 phase and apoptosis relatively easily in QR1 and QR2 cells compared with MV4-11 cells.

**Figure 8:**
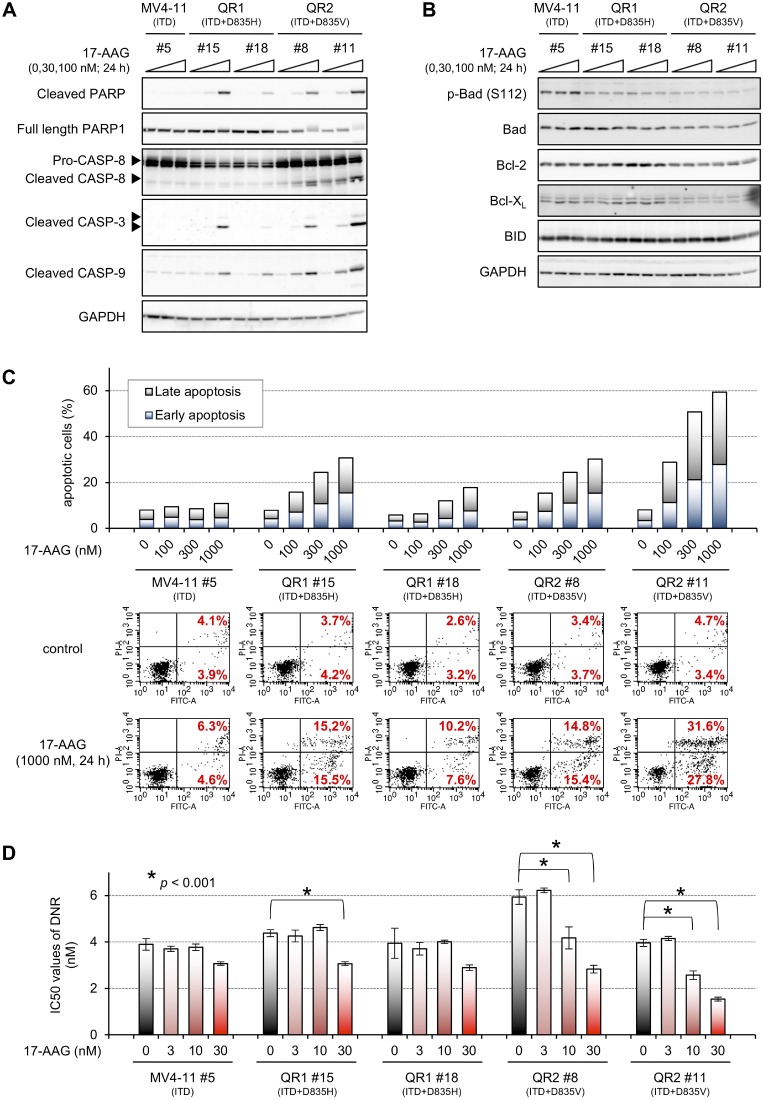
17-AAG-mediated apoptosis in QR1 and QR2 cells (**A** and **B**) Cells were treated with or without 30 or 100 nM 17-AAG for 24 h, and immunoblotting using the indicated antibodies was performed. Typical caspases were examined in (**A**), and mitochondrial apoptosis-related proteins were examined in (**B**). (**C**) Cells were treated with or without 100–1000 nM 17-AAG for 24 h and stained with annexin V-FITC and PI. The cells were then analyzed by FACS with quadrant statistics. The graphical data of all samples (top) and selected observed data (bottom) are shown. The corresponding raw data of all samples are shown in Supplementary Figure 6. (**D**) Cells were treated with increasing concentrations of daunorubicin (DNR, 0.25–64 nM) combined with or without 3–30 nM 17-AAG for 4 days, and the resulting cell viabilities were determined by WST-8 assay. The IC_50_ values of DNR are presented here as the mean ± SD from three independent experiments. Statistical analyses were performed with two-tailed Student's *t*-tests. Asterisks indicate *p* < 0.001, which was considered to be statistically significant. The corresponding raw data are shown in Supplementary Figure 7.

The cleavage of caspases and poly (ADP-ribose) polymerase (PARP) was examined in cells treated with 30 or 100 nM 17-AAG for 24 h (Figure 8A). 17-AAG induced cleavage of PARP and caspases-3 and -9 in QR1 and QR2 cells, and the cleavage was more obvious in QR2 cells than in QR1 cells, especially for caspases-8 and -9 (Figure 8A); however, the cleavage was dissociated with mitochondrial caspase activation (Figure 8B). Staining with annexin V-FITC and PI was then performed to examine the induction of apoptosis in 17-AAG-treated cells (Figure 8C and Supplementary Figure 6). The amount of apoptotic MV4-11 #5 cells barely increased following 17-AAG treatment. In contrast, 17-AAG induced apoptosis in QR1 and QR2 cells in a dose-dependent manner, particularly in QR2 #11 cells.

Finally, growth inhibition assays were conducted on cells treated with daunorubicin (DNR), which is an anticancer drug used in AML therapy, cultured in the presence of 17-AAG for 4 days (Figure 8D and Supplementary Figure 7). DNR-mediated growth inhibition was significantly enhanced by 30 nM 17-AAG in QR1 #15 cells, and co-treatment of 10 or 30 nM 17-AAG upregulated sensitivity to DNR in QR2 #8 and QR2 #11 cells. These results suggest that HSP90 inhibitors enhance sensitivity to DNR specifically in quizartinib-resistant cells, and HSP90 inhibitors have potential as drugs to overcome quizartinib resistance.

## DISCUSSION

Drug-resistance-conferring mutations of FLT3-ITD are serious problems in quizartinib treatment, and previous work has attempted to overcome quizartinib resistance through the use of other tyrosine kinase inhibitors, such as sorafenib and ponatinib [16–18]. Additionally, next-generation FLT3 inhibitors are under development, and phase studies of FF-10101 (Fujifilm) and gilteritinib (Astellas Pharma) are currently ongoing [13, 27, 28]. These inhibitors have been reported to possess good efficacy against the quizartinib resistance-conferring mutations of FLT3-ITD, including F691 and D835 mutations [13, 27, 28]. However, further strategies will still be required for AML therapy because our data reveal that Ba/F3-ITD+D835V and Ba/F3-ITD+F691L cells display an intermediate resistance and hyper-resistance, respectively, to gilteritinib (Figure 1B). Additionally, Ba/F3-ITD+F691L and Ba/F3-ITD+N676K cells also showed resistance to midostaurin (Figure 1B). The F691L mutation in FLT3-ITD confers resistance to all tested FLT3 inhibitors. In addition, based on prior clinical experience using other inhibitors in lung cancer (e.g., switching crizotinib to alectinib [29] or gefitinib to osimertinib [30]), AML cells will almost certainly acquire further resistance to the next-generation FLT3 inhibitors through additional mutations.

Here, we screened for inhibitors with different molecular actions against FLT3-ITD and detected HSP90 inhibitors as potential candidates (Figure 1). HSP90 inhibitors downregulated FLT3 signaling and induced apoptosis both in Ba/F3 transfectants (Figures 2–4; Tables 2 and 4; Supplementary Figures 1–4) and in QR1 and QR2 cells (Figures 7 and 8; Table 6; Supplementary Figures 5 and 6). HSP90 inhibitors are more efficacious in quizartinib-resistant cells than in quizartinib-sensitive cells. Specifically, Ba/F3-ITD+D835V, Ba/F3-ITD+F691L, Ba/F3-ITD+Y842C, QR1, and QR2 cells were all quizartinib-resistant but were also more sensitive to HSP90 inhibitors than their corresponding parental cells (Figures 1–4 and 6–8). There are two possible reasons for these results: (1) HSP90 inhibitors directly suppress FLT3 downstream signaling, including AKT, RAF, and/or STAT5; or (2) the inhibitors cause TKD mutants, particularly FLT3-ITD+D835V, to undergo more rapid proteolysis than FLT3-ITD. The first reason seems unlikely because 17-AAG, at the concentrations used in our experiments, did not affect the levels of phosphorylated STAT5, AKT, and ERK in parental Ba/F3 cells, whereas these levels were downregulated by 17-AAG in all transfectants (Figure 2B and 2C). To test a possible mechanism for the second potential reason, we examined proteolysis in 17-AAG-treated cells and found that BAFA1 treatment recovered the 17-AAG-mediated downregulation of FLT3-ITD+D835V but not FLT3-ITD (Figure 4A–4D). However, 17-AAG did not affect the binding of HSP90 to FLT3 *in vitro* (Figure 5B), although the binding decrease paralleled the reduced FLT3 expression observed in the cell-based assay (Figure 5A). HSP90 is a molecular chaperon complexed with p50^CDC37^ and p23, and it stabilizes newly synthesized proteins to ensure correct folding or refolds proteins that were damaged by various cellular stresses. HSP90 inhibitors promote conformational change and replace the binding partner for HSP90 [31, 32]. Our results suggest that HSP90 inhibitors may equally dissociate HSP90 bound to either FLT3-ITD or FLT3-ITD+D835V, but the resulting free FLT3-ITD+D835V would be destabilized more easily than FLT3-ITD during protein synthesis.

Another potential mechanism for the second explanation is that HSP90 inhibitors might change the affinity of ATP-binding to FLT3-ITD with or without TKD mutations. Therefore, we also determined the binding site of HSP90, which we identified as the region between TKD1 and TKD2 (Figure 5C). The 3D structure of the FLT3 kinase domain (PDB ID: 4XUF) reveals that the binding region is located opposite the activation loop, including D835 and Y842, corresponding to the entrance of the ATP-binding pocket (Supplementary Figure 8), indicating that HSP90-binding might conformationally control ATP-binding to FLT3. The replacement of the HSP90 complex by inhibitors might prohibit access of ATP to the binding pocket of the FLT3 kinase domain and achieve inactivation. Unlike other mutations, the substitution of Asp835 to Val would enhance the prohibition by steric hindrance. To confirm this hypothesis, further experiments, such as kinase assays and/or HSP90 binding assays with co-chaperones to FLT3-ITD+D835V compared with FLT3-ITD, conducted in the presence and absence of HSP90 inhibitors, will be required in future.

HSP90 inhibitors were previously shown to induce the proteasomal degradation of FLT3-ITD [33]. In contrast, proteasome inhibitors were reported to provoke FLT3-ITD degradation through autophagy [34], suggesting that unknown factor(s), the expression of which are partly regulated by proteasomal degradation, promote the autophagic protein digestion of FLT3-ITD. Our data support this mechanism because BTZ downregulated FLT3-ITD expression, whereas BAFA1 upregulated it (Figure 4A, compare lane 7 to lanes 9 or 11). Interestingly, BTZ did not change FLT3-ITD+D835V expression, whereas BAFA1 clearly increased protein expression and rescued 17-AAG-mediated downregulation of FLT3-ITD+D835V (Figure 4A). These results suggest a lost or reduced cooperation between the ubiquitin–proteasome system and the autophagy system in FLT3-ITD+D835V degradation; HSP90 inhibitors would promote the mutant protein to direct autophagic digestion regardless of the ubiquitin–proteasome pathway. Thus, the failure of normal degradation mechanisms might be one reason for Ba/F3-ITD+D835V cells having the highest sensitivity to HSP90 inhibitors.

Several groups have reported the relationship between HSP90 or HSP90 inhibitors with FLT3 [35–42], in which they examined the stability of FLT3-WT or FLT3-ITD without mutations in TKD. Yu *et al.* demonstrated that HSP90 inhibitors downregulate various TKD mutants, excluding D835 and Y842 mutations, of FLT3-ITD in mouse bone marrow 32D transfectants [33]. They also showed that HSP90 inhibitors downregulate the expression of D835Y mutants of FLT3-WT; however, they did not investigate D835Y mutants of FLT3-ITD. The present study focused on TKD mutations of FLT3-ITD, specifically F691, D835, and Y842, that confer resistance to three HSP90 inhibitors. This point differs from that of the report by Yu *et al.*, although similar FLT3-ITD+F691L results in quizartinib resistance were obtained in both studies. The cells expressing FLT3-ITD+F691L showed resistance to all tested FLT3 inhibitors (Figure 1), but they were sensitive to HSP90 inhibitors (Figure 2). These results, particularly those concerning gilteritinib treatment, are novel findings. Moreover, D835 mutation-harboring AML cells (QR1 and QR2 cells) were established in this study and used to assess the efficacy of HSP90 inhibitors. This work is the first to determine that, among known quizartinib-resistant mutants, FLT3-ITD+D835V-expressing cells are the most sensitive to HSP90 inhibitors.

Overall, HSP90 inhibitors overcome resistance to FLT3 inhibitors in Ba/F3 transfectants and quizartinib-resistant MV4-11 cells, particularly those with FLT3-ITD+D835V-mediated resistance. HSP90 is therefore a good molecular target for AML-related resistance to quizartinib and next-generation FLT3 inhibitors. To be prepared for the appearance of additional resistance mutations, HSP90 inhibitors should be developed for AML therapy.

## MATERIALS AND METHODS

### Reagents

Quizartinib, midostaurin, and gilteritinib were purchased from Selleck Chemicals (Houston, TX). 17-AAG, 17-DMAG, retaspimycin hydrochloride, and propidium iodide (PI) were obtained from Alomone Labs (Jerusalem, Israel), APExBIO (Houston, TX), and Wako (Tokyo, Japan), respectively. Daunorubicin (DNR) was from Sigma-Aldrich (St. Louis, MO). All other inhibitors were obtained from their original developers.

### Plasmids

The *FLT3*-ITD gene was amplified by conventional polymerase chain reaction (PCR) method using the cDNA library isolated from human AML cell line MV4-11 as a template. The addition of a *FLAG* sequence to the 3′-termini of *FLT3*-ITD genes was generated by PCR with *FLAG* sequence-conjugating *FLT3* primers. The *FLT3*-ITD-FLAG cDNA was cloned into a pCR2.1 vector (Invitrogen, Carlsbad, CA). The plasmid was digested with *Not*I and *Bam*HI followed by ligation of *FLT3*-ITD-FLAG to the pQCXIP vector (TaKaRa Bio, Ohtsu, Japan), which encodes a puromycin resistance gene. Substitution was performed using a QuikChange II Site-Directed Mutagenesis Kit (Agilent Technologies, Santa Clara, CA), following the manufacturer's instructions.

### Cells

The mouse pro-B cell line Ba/F3 was provided by the RIKEN BRC (Tsukuba, Japan) through the National Bio-Resource Project of the MEXT, Japan. The cells were maintained in RPMI-1640 medium supplemented with 10% fetal bovine serum (FBS), 10 ng/mL mIL-3, and 50 μg/mL kanamycin at 37° C in 5% CO_2_. Ba/F3 cells expressing FLT3-ITD or TKD mutations were established by retroviral transduction as described previously [43]. The transfectants were maintained in RPMI-1640 medium supplemented with 10% FBS, 50 μg/mL kanamycin, and 0.5 μg/mL puromycin. MV4-11 cells were obtained from the American Type Culture Collection (Manassas, VA) and maintained in IMDM medium supplemented with 10% FBS and 50 μg/mL kanamycin. The quizartinib-resistant MV4-11 cell lines QR1 and QR2 were independently established by culturing cells in the presence of increasing concentrations of quizartinib for 6 months. Quizartinib-resistant cells were maintained in IMDM containing each final concentration of quizartinib. The media were changed to quizartinib-free IMDM 3–4 days before starting experiments.

### Cell growth inhibition assay

Cell growth inhibition assays were performed via 4-[3-(2-methoxy-4-nitro-phenyl)-2-[4-nitrophenyl]-2H-5-tetrazolio]-1,3-benzene disulfonate sodium salt (WST-8)-based assays (Cell Counting kit-8, DOJINDO Laboratories, Kumamoto, Japan), and the IC_50_ values were determined from the growth inhibition curves, as described previously [44, 45]. Data represent the mean ± SD from triplicate experiments. The clustered image map was created at the CIMminor site (https://discover.nci.nih.gov/cimminer/home.do) using average IC_50_ values from three independent experiments.

### Immunoprecipitation and immunoblotting

Immunoprecipitation and immunoblotting were performed as described previously [43–45]. The following antibodies were used for immunoblotting: peroxidase-conjugated anti-FLAG M2 antibody (Sigma-Aldrich); peroxidase-conjugated anti-HA antibody (3F10; Roche Applied Science, Penzberg, Germany); anti-glyceraldehyde-3-phosphate dehydrogenase (GAPDH) antibody (6C5; Merck Millipore, Billerica, MA); anti-p53, anti-p27, anti-p21, and anti-PARP1 antibodies (Santa Cruz Biotechnology, Santa Cruz, CA); anti-cyclinA antibody (BD Biosciences, San Jose, CA); anti-cleaved PARP (Promega, Madison, WI); and other antibodies (Cell Signaling Technology, Danvers, MA).

### Flow cytometric analyses

To evaluate cell cycle ploidy patterns, cells were harvested after drug treatment, fixed with 70% ethanol, stained with PI solution (200 μg/mL RNase A and 50 μg/mL PI in PBS), and subjected to fluorescent-activated cell sorting (FACS) (FACS Calibur flow cytometer, BD Bioscience). To assess apoptotic cells, Annexin V-FITC and PI staining was performed using an Annexin V-PI staining kit (Roche Applied Science), following the manufacturer's instructions. Cells were subjected to FACS (BD LSR II flow cytometer, BD Biosciences). Quantitative data were analyzed using CellQuest software (BD Bioscience).

### Immunofluorescence staining

Cells were treated with or without 300 nM 17-AAG and/or 100 nM BAFA1 for 6 h, and 1 × 10^6^ cells were collected into 1.5 mL microcentrifuge tubes by centrifugation at 200 × *g* for 5 min at 4° C. After washing cells with ice-cold PBS, they were fixed with 3.7% formalin/PBS for 15 min at room temperature, permeabilized with 0.2% Triton X-100/PBS for 15 min at room temperature, transferred to FBS-coated 0.2-mL PCR tubes, and then blocked in 3% BSA/PBS for 30 min at room temperature. Cells were incubated with anti-FLAG M2 (50 μg/mL, Sigma-Aldrich) and anti-LC3 (1:50 dilution, Cell Signaling Technology) antibodies overnight at 4° C, followed by incubation with secondary antibodies, Alexa Fluor^®^ 488 goat anti-mouse IgG and Alexa Fluor^®^ 594 anti-rabbit IgG antibodies (Invitrogen), for 2 h at room temperature. Cells were washed three times with PBS, plated on cytoslides (Thermo Fisher Scientific Inc., Waltham, MA) using Cytospin^®^4 (Thermo Fisher Scientific Inc.), and mounted with Prolong^®^ Gold antifade reagent with DAPI (Invitrogen). Acquisition of images was performed using a FV1000-D IX81 confocal microscope (Olympus Corp., Tokyo, Japan). Confocal 2-D TIFF images were merged using Adobe^®^ Photoshop CC software (Adobe Systems Inc., San Jose, CA).

## SUPPLEMENTARY MATERIALS FIGURES AND TABLES



## Abbreviations

HSP90: Heat shock protein 90
FLT3: Fms-like tyrosine kinase 3
AML: acute myeloid leukemia
STAT5: signal transducer and activator of transcription 5
MAPK: mitogen-activated protein kinase
WT: wildtype
ITD: internal tandem duplication
TKD: tyrosine kinase domain
mIL-3: mouse interleukin-3
ERK: extracellular signal-regulated kinase
17-AAG: 17-allylamino-17-demethoxygeldanamycin
17-DMAG: 17-dimethylaminoethylamino-17-demethoxygeldanamycin
BAFA1: bafilomycin A1
BTZ: bortezomib
PARP: poly (ADP-ribose) polymerase
DNR: daunorubicin
