# Supplementary material for: Heat shock protein 90 inhibitors overcome the resistance to Fms-like tyrosine kinase 3 inhibitors in acute myeloid leukemia
Source: Oncotarget. 2018 Sep 28;9(76):34240–58. doi: 10.18632/oncotarget.26045 (PMC6188142; doi:10.18632/oncotarget.26045)
Supplement: Supplementary file 1 [file oncotarget-09-34240-s001.pdf]

Heat shock protein 90 inhibitors overcome the resistance to Fms-like tyrosine kinase 3 inhibitors in acute myeloid leukemia

SUPPLEMENTARY MATERIALS

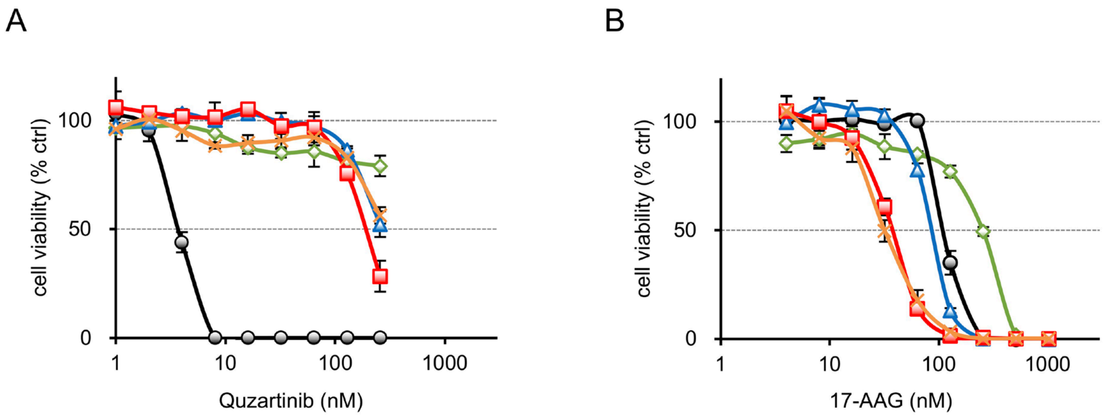

		IC_{50} (nM)	RR (vs ITD)
	Ba/F3+IL-3	> 256	
	Ba/F3-ITD	3.77	1.0
	Ba/F3-ITD+F691L	> 256	> 68
	Ba/F3-ITD+D835V	198	53
	Ba/F3-ITD+D835V-2	> 256	> 68

		IC_{50} (nM)	RR (vs Ba/F3)
	Ba/F3+IL-3	254	1.0
	Ba/F3-ITD	113	0.44
	Ba/F3-ITD+F691L	91.5	0.36
	Ba/F3-ITD+D835V	39.4	0.16
	Ba/F3-ITD+D835V-2	31.9	0.13

Supplementary Figure 1: Ba/F3-ITD+D835V-2 cells (not a clone of the former cell line) were established independently with same methods used to generate the previous cell line. (**A**) Cells were treated with increasing concentrations of quizartinib (1–256 nM) for 4 days, and cell viabilities were determined by WST-8 assay. The data are presented here as the mean ± SD from three independent experiments. The relative resistance (RR) was calculated by dividing the IC_{50} values of each cell line by that of Ba/F3-ITD cells. (**B**) Cells were treated with increasing concentrations of 17-AAG (4–1024 nM) for 4 days, and cell viabilities were determined by WST-8 assay. The data are presented here as the mean ± SD from three independent experiments. The RR was calculated by dividing the IC_{50} values of each cell line by that of Ba/F3 cells.

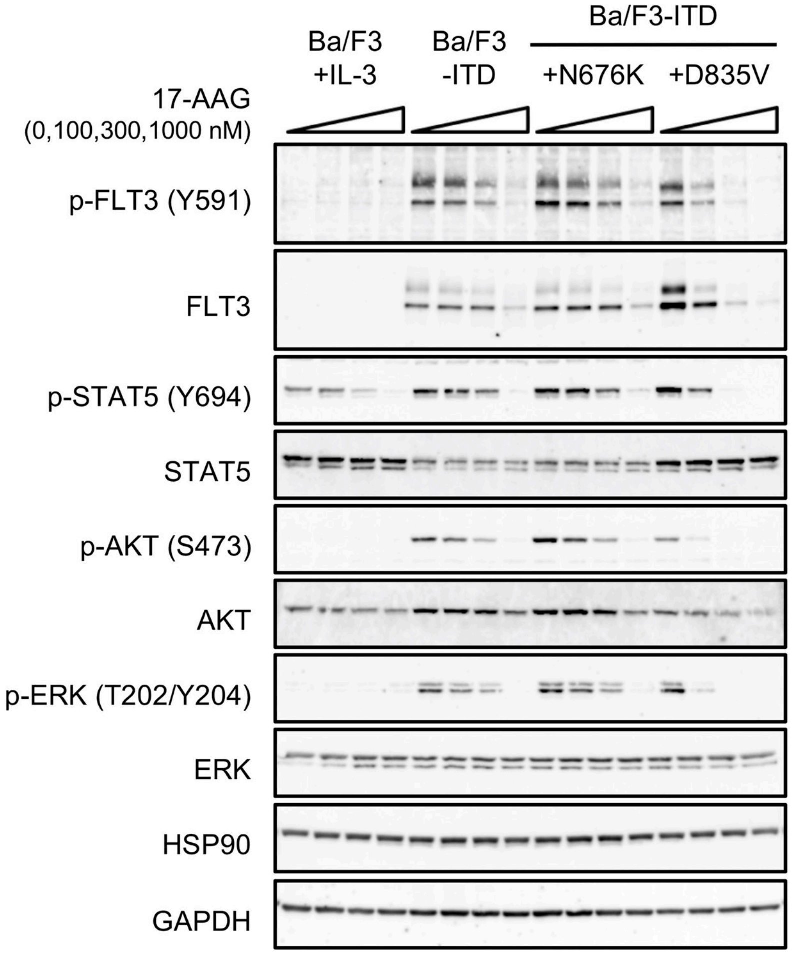

Supplementary Figure 2: The effect of 17-AAG on FLT3 signaling was assessed in limited cell lines to determine the appropriate drug concentrations. Cells were treated without or with 17-AAG at concentrations of 100, 300, or 1000 nM for 6 h. Ba/F3 cells were treated with the drug in the presence of 10 ng/mL_ IL-3 (+IL-3). Cell lysates were prepared and subjected to immunoblots using the indicated antibodies.

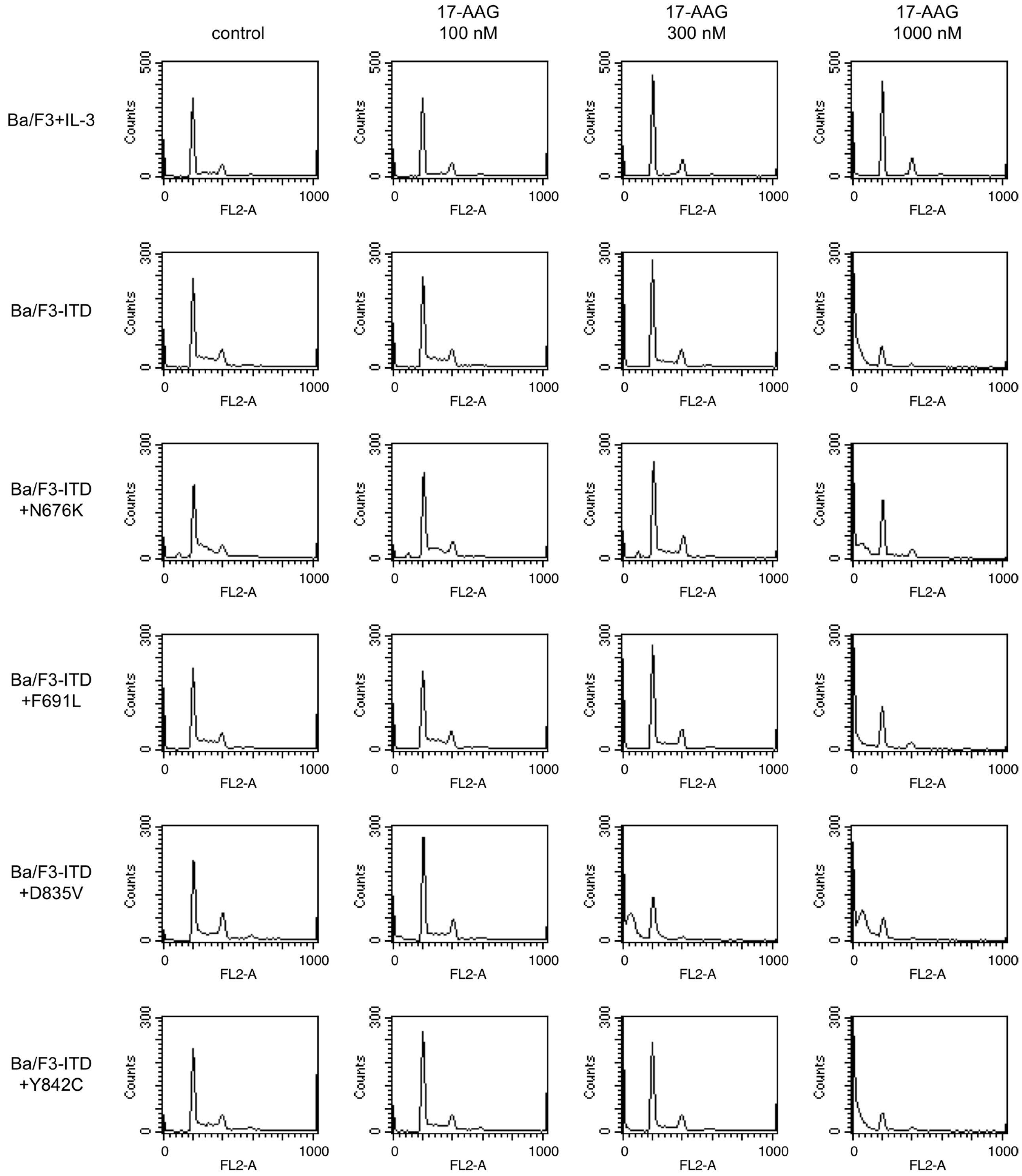

Supplementary Figure 3: Cells were treated with the indicated concentrations of 17-AAG (100, 300, or 1000 nM) for 24 h, and their ploidy patterns were determined by flow cytometry after staining with 50 µg/mL propidium iodide containing 1 pg/mL RNase for 30 min at room temperature in the dark. A summary of these results is shown in Figure 2D.

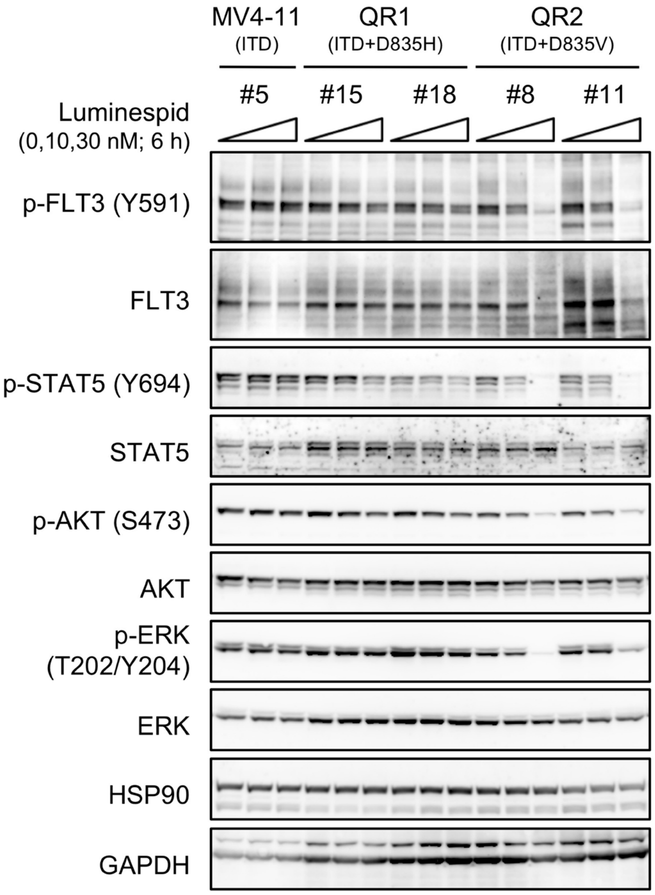

Supplementary Figure 4: The effect of luminespid on FLT3 signaling was assessed in MV4-11 #5 and the quizartinib-resistant cells, QR1 (#15 and #18) and QR2 (#8 and #11). Cells were treated without or with luminespid at concentrations of 10 or 30 nM for 6 h. Cell lysates were prepared and subjected to immunoblots using the indicated antibodies.

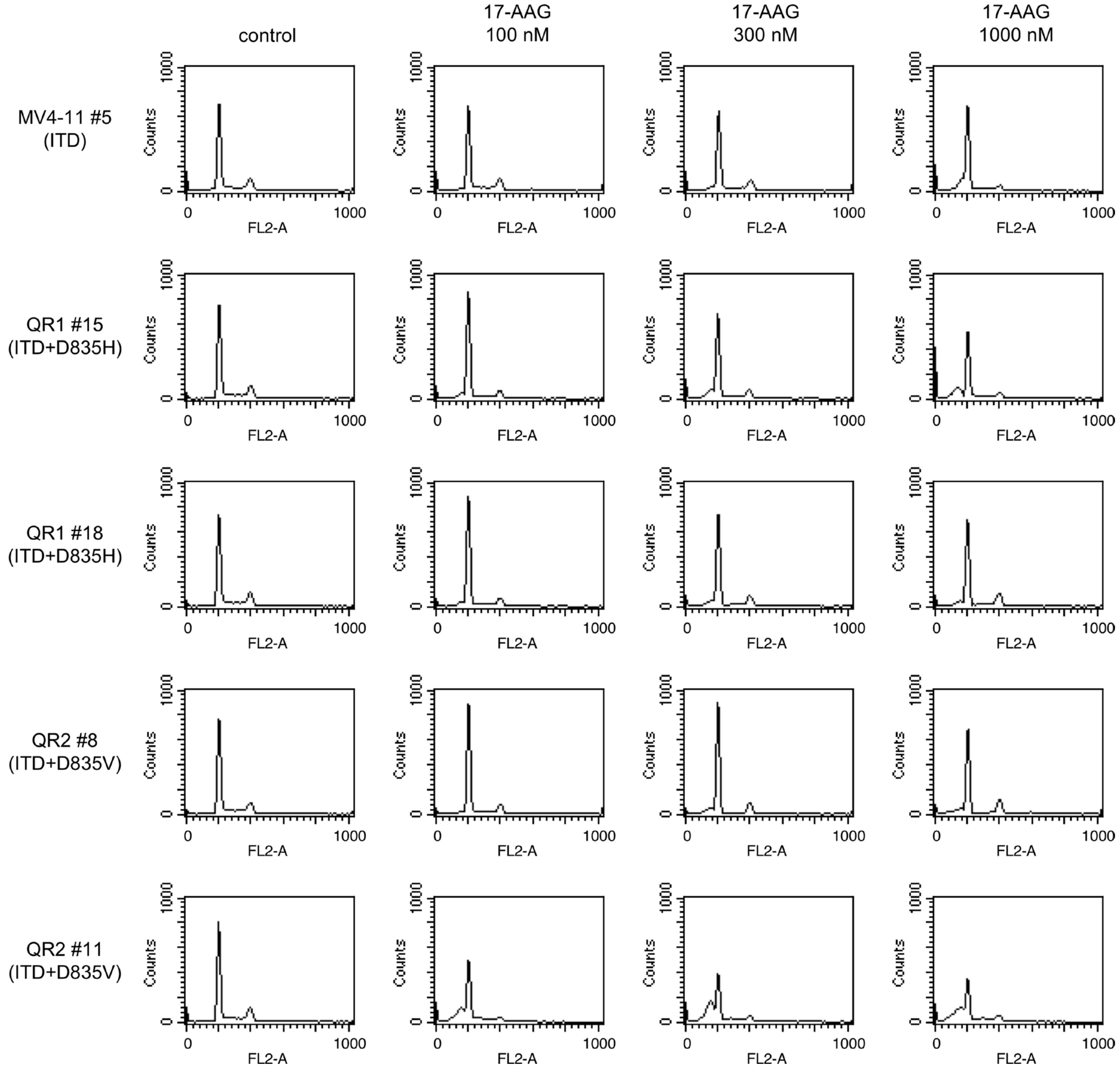

Supplementary Figure 5: Cells were treated with the indicated concentrations of 17-AAG (100, 300, or 1000 nM) for 24 h, and their ploidy patterns were determined by flow cytometry after staining with 50 μg/mL propidium iodide containing 1 pg/mL RNase for 30 min at room temperature in the dark. A summary of these results is shown in Figure 6D.

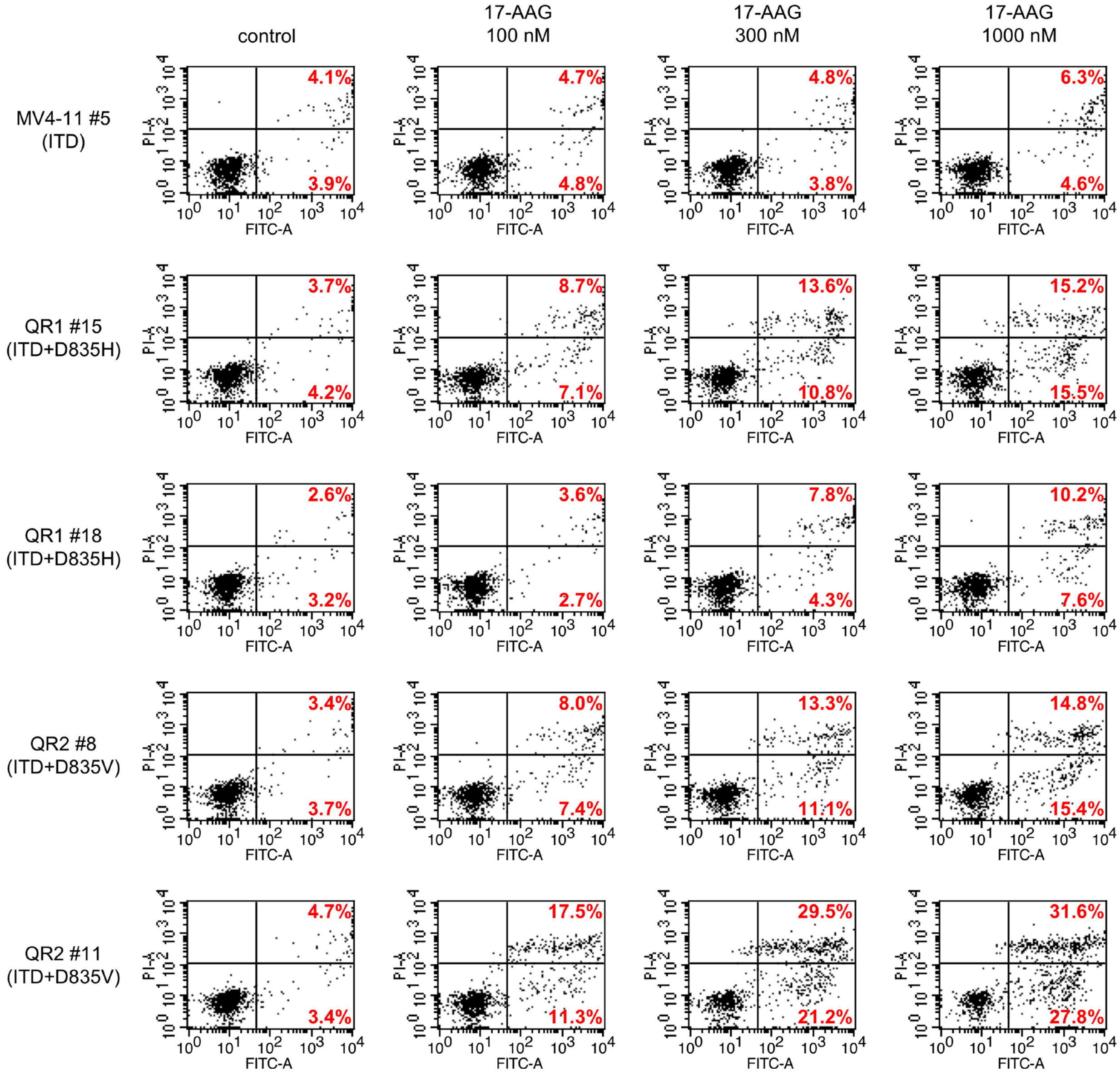

Supplementary Figure 6: Cells were treated with the indicated concentrations of 17-AAG (100, 300, or 1000 nM) for 24 h. Cells were stained with Annexin V-FITC and PI, and apoptotic cells were quantified by flow cytometry. A summary and selected data from these results are shown in Figure 7C.

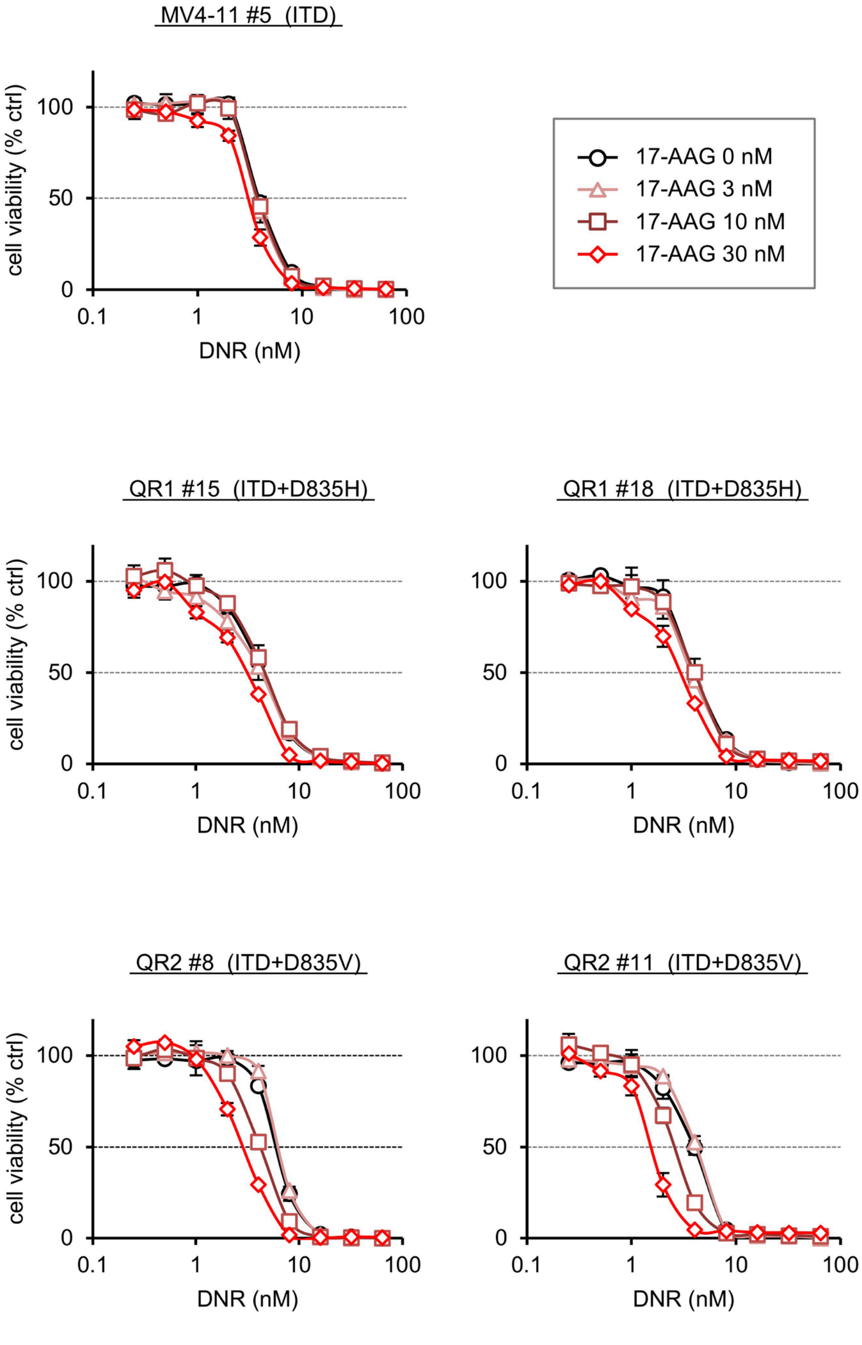

Supplementary Figure 7: MV4-11, QR1, and QR2 cells were treated with increasing concentrations of daunorubicin (DNR, 0.25–64 nM) combined with or without 3, 10, or 30 nM 17-AAG for 4 days, and cell viabilities were determined by WST-8 assay. The data are represented here as the mean ± SD from three independent experiments. A summary of these results is shown in Figure 7D.

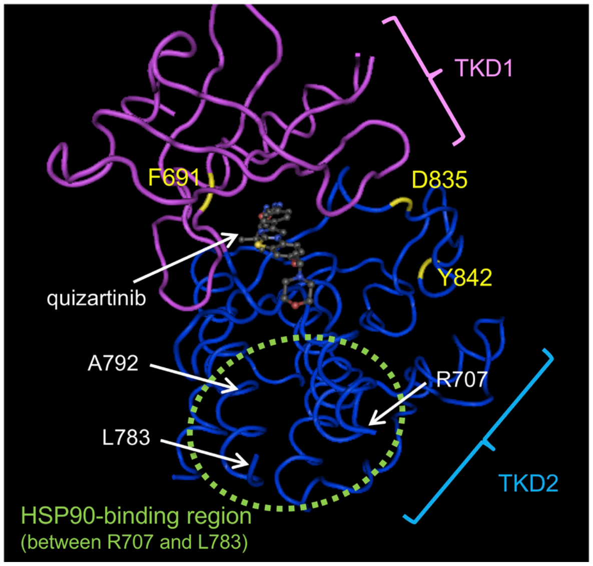

Supplementary Figure 8: The HSP90-binding area is indicated by a green dotted circle in the crystal structure of the FLT3 kinase domain bound to quizartinib (PDB ID: 4XUF); the region between T707 and L783 is disconnected. The region between amino acids 721–792 is prospective to the binding site from the result of Figure 4C. The site is conformationally located opposite the activation loop, including D835 and Y842, corresponding to the entrance of the ATP-binding pocket.

Supplementary Table 1: Inhibitors used in drug screening (Figure 1D)

Targets	Inhibitors
AKT	AZD5363
ALK, c-MET	crizotinib
ATM/ATR	KU-55933
AURK	tozasertib (VX-680)
B-RAF	GDC-0879
BCR-ABL, c-KIT	nilotinib
BCR-ABL, c-KIT	imatinib
BCR-ABL, Src	dasatinib
BRD	(+)-JQ1
C-RAF	ZM336372
Ca ionophore	A23187
CDC34	CC0651
CDK4/6	palbociclib
COX-2	NS-398
DNMT	azacitidine
EGFR	gefitinib
EGFR	erlotinib
FLT3	quizartinib
HDAC	vorinostat
HER2, EGFR	lapatinib
HSP90	tanespimycin (17-AAG)
HSP90	alvespimycin (17-DMAG)
IR, IGF-1R	BMS-536924
JAK	tofatinib
JNK	SP600125
MEK1/2	trametinib
mTORC1	everolimus
multi-kinases	pazopanib
multi-kinases	sorafenib
multi-kinases	axitinib
multi-kinases	sunitinib
N-linked glycosylation	tunicamycin
p38MAPK	PD169316
PARP	olaparib
PDE5 (cGMP)	zaprinast
PI3K	ZSTK474
PI3K	pictilisib (GDC-0941)
PI3K, mTOR	dactolisib (NVP-BEZ235)
PIM	SGI-1776
PKA	forskolin
PKC	enzastaurin
PKC	sotrastaurin
PLK1	volasertib (BI 6727)
PP1	PP1 analog
PP2A	cantharidin
proteasome	bortezomib
RSK	BI-D1870
VEGFR, EGFR, RET	vandetanib
VEGFR, PDGFR	vatalanib
VEGFR2, c-MET	foretinib (XL-880)

The list is alphabetically displayed in targets.
